# Effects of Airgun Sounds on Bowhead Whale Calling Rates: Evidence for Two Behavioral Thresholds

**DOI:** 10.1371/journal.pone.0125720

**Published:** 2015-06-03

**Authors:** Susanna B. Blackwell, Christopher S. Nations, Trent L. McDonald, Aaron M. Thode, Delphine Mathias, Katherine H. Kim, Charles R. Greene, A. Michael Macrander

**Affiliations:** 1 Greeneridge Sciences, Inc., Santa Barbara, California, United States of America; 2 Western EcoSystems Technology, Inc., Cheyenne, Wyoming, United States of America; 3 Scripps Institution of Oceanography, La Jolla, California, United States of America; 4 Shell Exploration & Production Company, Anchorage, Alaska, United States of America; Pacific Northwest National Laboratory, UNITED STATES

## Abstract

In proximity to seismic operations, bowhead whales (*Balaena mysticetus*) decrease their calling rates. Here, we investigate the transition from normal calling behavior to decreased calling and identify two threshold levels of received sound from airgun pulses at which calling behavior changes. Data were collected in August–October 2007–2010, during the westward autumn migration in the Alaskan Beaufort Sea. Up to 40 directional acoustic recorders (DASARs) were deployed at five sites offshore of the Alaskan North Slope. Using triangulation, whale calls localized within 2 km of each DASAR were identified and tallied every 10 minutes each season, so that the detected call rate could be interpreted as the actual call production rate. Moreover, airgun pulses were identified on each DASAR, analyzed, and a cumulative sound exposure level was computed for each 10-min period each season (*CSEL_10-min_*). A Poisson regression model was used to examine the relationship between the received *CSEL_10-min_* from airguns and the number of detected bowhead calls. Calling rates increased as soon as airgun pulses were detectable, compared to calling rates in the absence of airgun pulses. After the initial increase, calling rates leveled off at a received *CSEL_10-min_* of ~94 dB re 1 μPa^2^-s (the lower threshold). In contrast, once *CSEL_10-min_* exceeded ~127 dB re 1 μPa^2^-s (the upper threshold), whale calling rates began decreasing, and when *CSEL_10-min_* values were above ~160 dB re 1 μPa^2^-s, the whales were virtually silent.

## Introduction

Marine mammals rely heavily on both hearing and producing sounds for prey detection, predator avoidance, mate selection, communication, navigation, and other important life-history functions. Worldwide increases in underwater sound levels of anthropogenic origin [1–5] have been changing ocean acoustic environments for decades. Concern over how marine mammals are affected by and cope with these man-made sounds has motivated research on sound exposure thresholds that trigger biologically significant behavioral responses in various species. Some studies detected no behavioral changes in response to man-made sound [6, 7]. Others have shown changes in calling behavior [8–10], migratory pathway [11], or diving behavior [12] in response to sound stimuli. Recently, changes in calling behavior in response to low levels of sound received from distant sound sources has also been demonstrated in blue and humpback whales [13, 14].

Airgun pulses from seismic surveys are one of the main sounds of concern in the ocean environment because their low frequencies and high amplitudes allow them to travel over large distances when propagation conditions are favorable [15]. They are generally produced at 4–20 s intervals, over periods of days, weeks or months, albeit not continuously. For example, seafloor recorders in the Atlantic have detected airgun pulses on more than 80% of days over periods of several months [16]. Decreasing summer ice coverage at high latitudes over the past decade has opened up certain areas of the Arctic to increased oil and gas exploration. Some baleen whales, such as bowhead whales, are long-lived and migrate over long distances. These combined factors mean that over their lifetimes they are likely subjected to many airgun pulses. There is current interest in assessing cumulative effects of anthropogenic sounds on marine mammals over long time periods [17]—in a fashion similar to the studies done on humans [18]. For such assessments, information on behavioral reactions to airgun sounds are of vital importance, as they are common sound sources in ocean basins today.

For bowhead whales (*Balaena mysticetus*), the species of interest in this study, Blackwell *et al*. [19] showed calling rates decreased when whales were relatively close (median distance 41–45 km) to an operational airgun array. Median received airgun pulse levels (in terms of rms SPL) at those sites were at least 116 dB re 1 μPa. In contrast, whales that were relatively distant from the same operation (median distance >104 km), and received median airgun pulse levels below 108 dB re 1 μPa, did not change their calling rates. This raised the following question: At what received “dose” of sound did calling behavior change? The present study attempts to pinpoint thresholds of received sound levels from airgun pulses at which whale behavior changes.

In 2007, 2008, and 2010 Shell Offshore Inc. (Shell) conducted vessel—based seismic surveys and shallow hazard surveys on or near lease holdings in the Beaufort Sea. As part of these exploration activities, a passive acoustic monitoring program was implemented, with the objective of addressing the interaction of bowhead whales with industrial activities during their fall migration. This study is based on the data collected during passive acoustic monitoring efforts spanning the open-water seasons of 2007, 2008, 2009, and 2010. Two behavioral thresholds were identified in the response of bowhead whales to airgun activity: (1) At low received levels of airgun sound, the animals’ calling rates actually *increased* over baseline levels, but (2) when received levels exceeded a certain threshold, calling rates *decreased* rapidly.

## Methods

### Equipment

The equipment and field methods are the same as those presented in Blackwell *et al*. [19]. Recordings were made using Directional Autonomous Seafloor Acoustic Recorders (DASARs, model C, see [20]). DASARs include an omnidirectional calibrated hydrophone (sensitivity -149 dB re 1 V/μPa at 100 Hz; noise floor, in dB re 1 μPa^2^ / Hz: 62 dB @ 10 Hz, 48 dB @ 50 Hz, 44 dB @ 100 Hz, 37 dB @ 400 Hz), used for sound pressure measurements of the background sound field, including whale calls and airgun pulses. DASARs also include two particle motion sensors mounted orthogonally in the horizontal plane for sensing direction to sounds of interest, such as whale calls or airgun pulses. A 1 kHz sampling rate was used for each of these three data channels. The recorders included a signal digitizer with 16-bit quantization. Samples were buffered for about 45 min, then written to an internal 60 GB hard drive. Allowing for anti-aliasing, the 1 kHz sampling rate allowed for 116 days of continuous recording in the frequency range 10–450 Hz across the four years.

The hydrophone recorder electronics in the DASARs overloaded (saturated and distorted) when the instantaneous sound pressure (0-to-peak) exceeded 151 dB re 1 μPa at 100 Hz. This occurred with some of the received airgun pulses discussed below.

### Field Procedures

**Fig 1:**
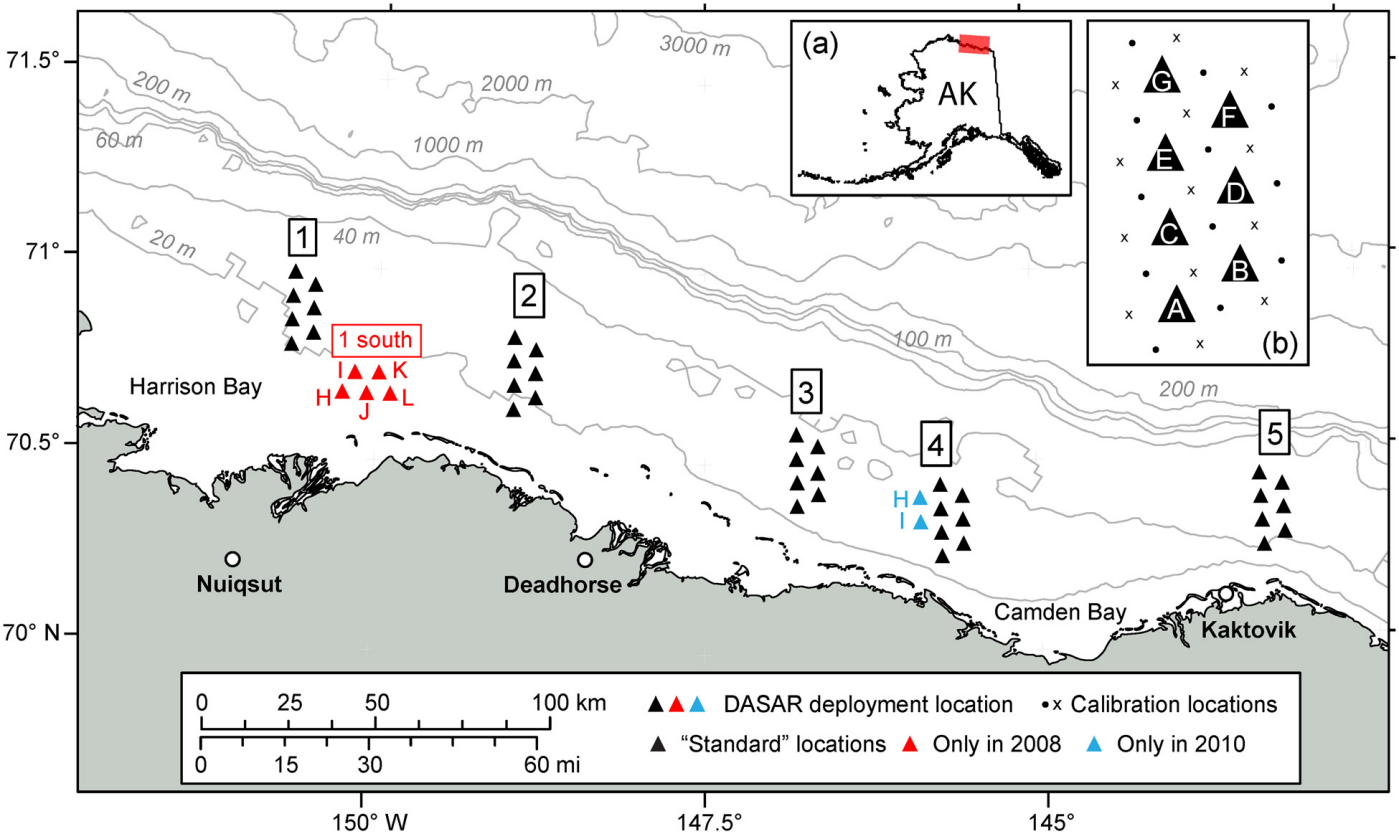
DASAR deployment locations used in the 2007–2010 field seasons. Inset (a) shows the location of the arrays on the map of Alaska. The five main, seven-DASAR arrays (black triangles), labeled 1–5 from west to east, were deployed each year, conditions permitting (ice prevented some deployments in 2010, see Table 1). DASARs were labeled A–G from south to north, as shown in inset (b). Other locations were used only in some years. In 2008 five recorders were deployed south of site 1: DASAR locations 1H, 1I, 1J, 1K, and 1L (red triangles). In 2010 two recorders were deployed west of site 4: DASAR locations 4H and 4I (blue triangles). Inset (b) shows calibration locations with respect to the DASARs’ locations at a single array. The same relative calibration locations were used at each site.

Each year during 2007–2010 up to 40 DASARs were deployed in the Beaufort Sea offshore of Alaska’s North Slope, spread over an alongshore distance of ~280 km. DASARs were deployed in five groups (“sites”), each comprised of 7–12 recorders, as shown in Fig 1. DASARs at each site were placed at the vertices of adjacent equilateral triangles with 7 km sides, and were labeled with letters (Fig 1, inset (b)). The southernmost DASARs were 15–33 km due north of the coast. Each DASAR was placed on the seafloor with a ground line of length 110 m connecting it to a small Danforth anchor. During deployment GPS positions were obtained for the DASAR and its anchor. Deployments in all years took place between August 6 and 26. Table 1 summarizes deployment information in all four years of the study. Water depths at deployment locations were in the range 15–53 m. The mean water depth of each seven-DASAR array (i.e., black triangles in Fig 1) increased from west to east and was 21.3 m, 26.7 m, 35.0 m, 35.4 m, and 48.1 m for sites 1, 2, 3, 4, and 5, respectively. DASAR deployment coordinates and water depths for all locations are given in S1 Table.

**Table 1:** Deployment and retrieval dates at all sites, number of records obtained, and days of useable data.

Year	Site	Deployment date	Retrieval date	Number of DASARs deployed	Number of usable records	Omitted records	DASAR-days of usable data
**2007**	1	24 Aug.	12 Oct.	7	5	1G, 1B	
	2	23 Aug.	11 Oct.	7	7		
	3	23 Aug.	8 Oct.	7	7		
	4	22 Aug.	10 Oct.	7	7		
	5	21 Aug.	9 Oct.	7	6.5	5D (1^st^ half)	
			2007 totals:	35	32.5		1570
**2008**	1	11^a^ & 17 Aug.	7^a^ & 8 Oct.	12	9	1F, 1L, 1B	
	2	16 Aug.	7 Oct.	7	7		
	3	21 Aug.	6 Oct.	7	6	3E	
	4	20 Aug.	5 Oct.	7	5	4A, 4D	
	5	19 Aug.	2 Oct.	7	7		
			2008 totals:	40	34		1637
**2009**	1	26 Aug.	4 Oct.	7	6	1G	
	2	25 Aug.	5 Oct.	7	7		
	3	23 Aug.	1 Oct.	7	6	3G	
	4	20 Aug.	2 Oct.	7	7		
	5	21 Aug.	5 Oct.	7	7		
			2009 totals:	35	33		1371
**2010**	1	6 Aug.	30 Sep.	7	7		
	2	7 Aug.	28 Sep.	2^b^	0	2F, 2G	
	3	13 Aug.	1 Oct.	6^c^	5	3F	
	4	12 Aug.	3 Oct.	9	9		
	5	11 Aug.	4 Oct.	7	6	5F	
			2010 totals:	31	27		1425
			**Overall totals:**	**141**	**126.5**	**14.5**	**6003**

The column “omitted records” includes instruments that were lost (e.g., 1G in 2007 or 5F in 2010), that experienced problems with bearing calibrations (e.g., 2F or 2G in 2010), or that failed to record (e.g., 1B in 2007 or 3F in 2010). Usable records at a site begin with the deployment of the site’s last DASAR. Similarly, usable records end with the retrieval of the site’s first DASAR.

^a^ Dates for the southern five-DASAR array deployed south of site 1 in 2008 (see Fig 1).

^b^ DASARs 2A, 2B, 2C, 2D, and 2E could not be deployed in 2010 because of ice.

^c^ DASAR 3A could not be deployed in 2010 because of ice.

After deployment, each DASAR’s orientation on the seafloor with respect to true north was determined in order to estimate bearing to a sound source. In addition, each DASAR clock experiences a small and constant drift, which was corrected over the course of a lengthy deployment period in order to time-align the DASARs [20]. Therefore, immediately following deployments and preceding retrievals, calibrations signals (source level ~150 dB re 1 μPa @ 1 m, frequency range 200–400 Hz) were transmitted at known GPS-determined times and locations: six (in 2007–2009) or three (in 2010) locations about ~4 km from each DASAR (see inset (b) in Fig 1; 2010 calibration locations shown with black dots). For more information on calibration methodology, see Greene *et al*. [20].

Retrieval was accomplished by grappling for the ground line, using the GPS positions obtained during deployment. DASARs were retrieved each year between September 28 and October 12 (Table 1), shortly before the Beaufort Sea begins freezing over. This deployment period captured much of the bowhead whale autumn migration [21], but not the tail end, which continues into late October or early November when boat-based operations are no longer possible due to the presence of sea ice.

### Permitting

Passive acoustic recording of endangered bowhead whale calls does not typically require a federal permit as it does not have the potential to “take” the animals as defined by the U.S. Marine Mammal Protection Act or the U.S. Endangered Species Act. The research presented here was, however, part of an approved monitoring program around activities conducted under incidental harassment authorizations (IHAs) issued by the U.S. National Marine Fisheries Service. The research was therefore subject to regulatory review and approval under those authorizations and under the terms of lease agreements under the Outer Continental Shelf Lands Act.

### Bowhead Call Detection and Localization

After retrieval, the DASARs’ housings were opened and their hard drives removed. Data were transferred to file servers and analyzed on workstations running custom MATLAB-based software. The data collected at each site and each year were analyzed with an automated call detection algorithm [22]. This analysis identified and localized bowhead calls and airgun pulses, as discussed further below. A subset of all data collected (100% in 2007, 12.5% in 2008, 19.3% in 2009, and 14.3% in 2010) was also analyzed manually by trained analysts as described in Blackwell *et al*. [23, 19]. These manually analyzed data served as a reference to which the automatically detected calls could be compared. The automated algorithm parameters (such as the neural network output threshold) were configured so that up to 20% of legitimate whale calls could be missed (recall of 0.8) in order to minimize false detection rates. An exact calculation of false detection rate was not possible because the manually analyzed data had significant biases and omissions. Nevertheless, the precision of the algorithm was estimated to be between 0.8 and 0.9 for a recall of 0.8. As a result, the automated detector always reported fewer bowhead calls than the manual analysts [22]. Further information on false detection rates is given in S1 File.

In addition to the whale call analysis described above, bearings to calling whales were determined for all DASARs at each of the five sites. When two or more DASARs at a given site detected the same call, the location of the calling whale was estimated using triangulation (crossfixing), as described in Greene *et al*. [20]. The Huber robust location estimator [24] was used to compute the location of each call, as well as the associated 90% confidence ellipse, based on the intersection(s) of bearings from all DASARs at a given site that detected the call [20, 19]. We define the call localization rate as the number of calls localized within a fixed period of time [19], distinct from both the number of calls detected (some of which are not localized) and the actual number of calls produced by whales (some of which are not detected).

### Defining the Analysis Area

**Fig 2:**
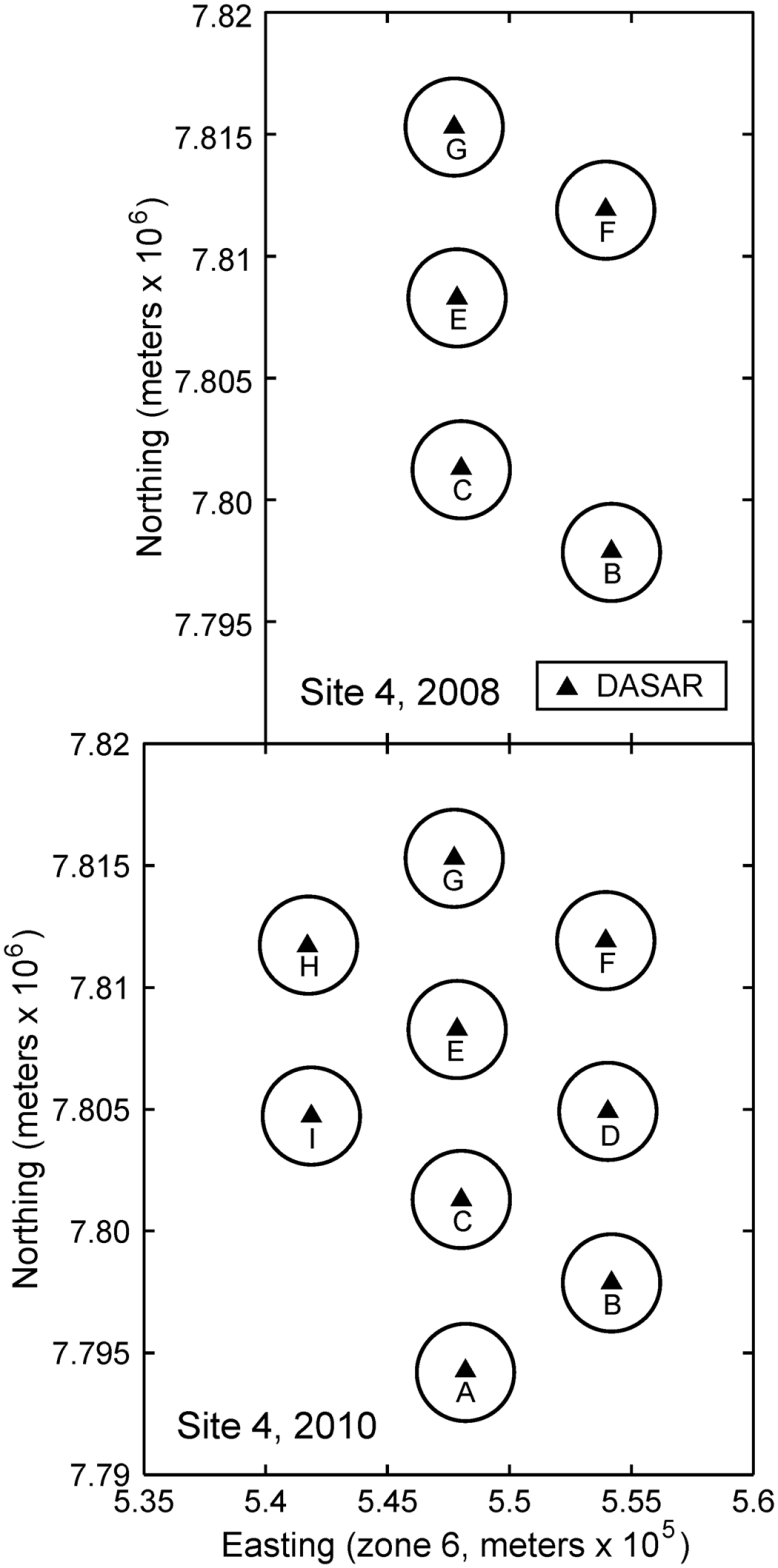
Examples of analysis cells for site 4 in 2008 (top) and 2010 (bottom). The analysis cells are circular areas of radius 2 km centered on the mean DASAR locations, shown with a black triangle. In 2008 records were not obtained at locations 4A and 4D (see Table 1 and Fig 1) so those cells are omitted from the figure. In 2010 all deployed DASARs at site 4 locations (A-I) yielded usable records.

The analysis was designed to identify a relationship between received levels of airgun sounds and bowhead whale calling behavior. The following two factors are therefore of critical importance: (1) all whale calls included in the analysis should have approximately the same probability of inclusion (i.e., detection), and (2) our estimate of the received levels (RLs) from airgun pulses must be accurate. To satisfy these two analysis requirements, we restricted our analysis area to a set of 42 “analysis cells”. Each analysis cell is a circle of radius 2 km centered on the mean location of each DASAR over the four years (variation in DASAR deployment locations was at most tens of meters between years). Examples of analysis cells are shown in Fig 2. Analysis cells in any given year were included if they represented a usable DASAR record.

The suitability of the 2 km size for the analysis cells, from the perspective of the first constraint (equal probability of detection), is presented in S2 File. With respect to the second constraint (accuracy of airgun pulse RLs), a main advantage of restricting the analyses to relatively small circular cells is that airgun pulse levels as measured at the DASAR central to each cell can be used as the “dose” of sound to which concurrent call detection rates are compared. Larger analysis areas would have required interpolation, statistical or numerical propagation modeling or other correction of received airgun pulse levels (see for example [19]) in areas away from the recorders. Meanwhile, the analysis cells are small enough that we are confident call localization rates at the DASARs are highly correlated with actual calling rates by the whales. Hereafter, when discussing calls detected inside the analysis cells we use “calling rate” as a proxy for “call localization rate”.

Another advantage of restricting the analyses to these cells is that it removes any possibility that distant dispersed airgun signals (which can display similar bandwidth and time-frequency structure to bowhead whale calls) could be mistaken by the automated algorithm for bowhead whale calls. This fact becomes important when interpreting estimated calling rates of whales at low signal received levels.

### Time Intervals

This analysis required defining the length of the time interval over which a dose (received level of sound from airgun pulses) and a response (whale calling rate) would be matched. The interval length needed to be long enough that many intervals included one or more calls. Conversely, the interval length needed to be short enough that potentially important variations in the received levels of airgun sounds over a time window would not be obscured. In addition, the interval length ought to be relevant for whale response to received levels of sound, of which little is known. Based on these considerations, we chose a time interval of 10 min and most of the results in this paper are presented for the 10-min interval. In addition, to test whether the results were sensitive to the choice of interval length, we conducted analyses for 5- and 20-min intervals. In each of the four years the entire field season (from DASAR deployment to retrieval) was partitioned into non-overlapping periods with lengths of 5, 10, or 20 min and which always began on the hour.

The number of whale calls localized within each analysis cell was tallied for each of the three time periods each year. Hereafter, a particular analysis cell at a particular time interval will be referred to as a “cell-time interval”. Over the four years, the following numbers of cell-time intervals were tallied: 1,704,688 (5 min), 852,344 (10 min), and 426,172 (20 min).

### Airgun Activity during 2007–2010

**Fig 3:**
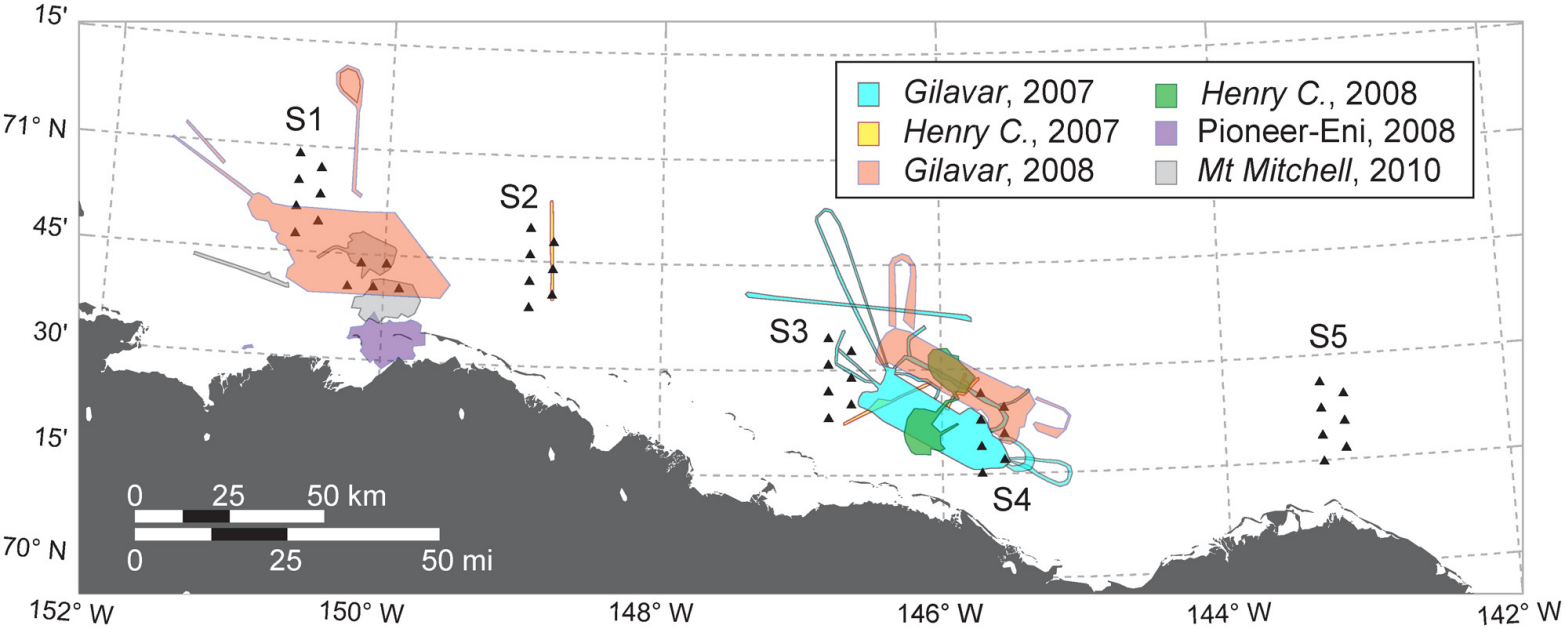
Locations of airgun use near the DASAR arrays in 2007, 2008, and 2010. Only operations that occurred either within the DASAR arrays or less than 50 km from the nearest DASAR are shown. (In 2009 there were no seismic exploration activities involving airguns in the area shown.) See Table 2 for dates of operation and array sizes.

There were a number of seismic exploration activities using airgun arrays in the Beaufort Sea in 2007–2010. Some of these activities were within the DASAR arrays, or a few km away, while others were hundreds of km away. We define “nearby” activities as those occurring less than 50 km from the nearest DASAR. Nearby activities, shown in Fig 3, were carried out by Shell in 2007, 2008, and 2010, and by PGS (under contract to Pioneer / Eni) in 2008. Dates of operation, vessels involved, and airgun array sizes of these activities are given in Table 2.

**Table 2:** Airgun operations taking place within or near the DASAR arrays in 2007–2010.

Year	Company	Vessel	Type	Max array volume (in^3^)	Dates
**2007**	Shell	*M/V Gilavar*	3-D surveys	3147	18 Sept.–3 Oct.
		*M/V Henry Christoffersen*	Shallow hazards	20	30 Aug., 14, 17, 18 Sept.
**2008**	Shell	*M/V Gilavar*	3-D surveys	3147	3 Sept.–9 Oct.
		*M/V Henry Christoffersen*	Shallow hazards	20	12–23 Aug.
	Pioneer / Eni	*M/V Wiley Gunner*, *M/V Shirley V*, and *M/V Peregrine*	Seismic and shallow hazard surveys	440 and 880	2 Aug.–26 Sept.
**2009**	No activities involving airguns near the DASAR arrays				
**2010**	Shell	*R/V Mt*. *Mitchell*	Shallow hazards	40	13, 16–19 Aug., 15–19, 21, 30 Sept., 1–6 Oct.

Information on the dates of operation, companies and vessels involved, survey type, and array volumes are presented.

“Distant” activities were carried out by various operators and were generally located several hundred km from the DASAR arrays. These activities included—but were not limited to—the Pokak 3-D Seismic Program (BP Canada East, Aug.–Oct. 2009, ~300–600 km E of our sites), the Canada Basin Seismic Reflection and Refraction Survey of the Western Arctic Ocean (Geological Survey of Canada and United States Geological Survey, Aug.–Sep. 2009 and 2010, ~300–1400 km N of our sites, see [25, 26]), and an offshore 2-D seismic program in the Canadian Beaufort (GXT, Aug.–Oct. 2010, at least 180–460 km E of our sites).

### Received Levels of Sounds from Airgun Pulses

To obtain a quantitative assessment of the number and received levels of airgun pulses detected at DASAR locations, we used an automated airgun pulse detector on every available DASAR record. This automated process utilized three stages [15, 22]. In the first stage a banded energy detector (constant false alarm rate) detected individual transient signals. The second stage used the regular inter-pulse intervals and azimuthal consistency that are characteristic of seismic exploration using airguns to discard pulses that were not produced by airguns. The third stage calculated the following six parameters (see [27, 28, 29], Appendix A in [30]) for each detected pulse: (1) “peak pressure”, i.e., the maximum of the received instantaneous sound pressures at the 1 ms sampling intervals (in dB re 1 μPa); (2) “duration”, defined as the time interval between the arrival of 5% and 95% of the total pulse energy (in s); (3) “sound pressure level” (SPL, rms), averaged over the pulse duration (dB re 1 μPa); (4) “sound exposure level” (SEL), a measure related to the energy in the pulse, defined as the squared instantaneous sound pressure integrated over the pulse duration (dB re 1 μPa^2^-s); (5) “background level”, the SPL measured over 0.5–1 s immediately preceding the pulse.; and (6) “bearing” (in °) from the DASAR to the airgun pulse. All metrics were computed after passing each transient time series through a finite-impulse response (FIR) bandpass filter, the details of which are explained in S3 File. The SPL and SEL estimates were obtained for “signal only”, i.e., after subtracting an estimate of the background noise level from the integrated measurement.

**Fig 4:**
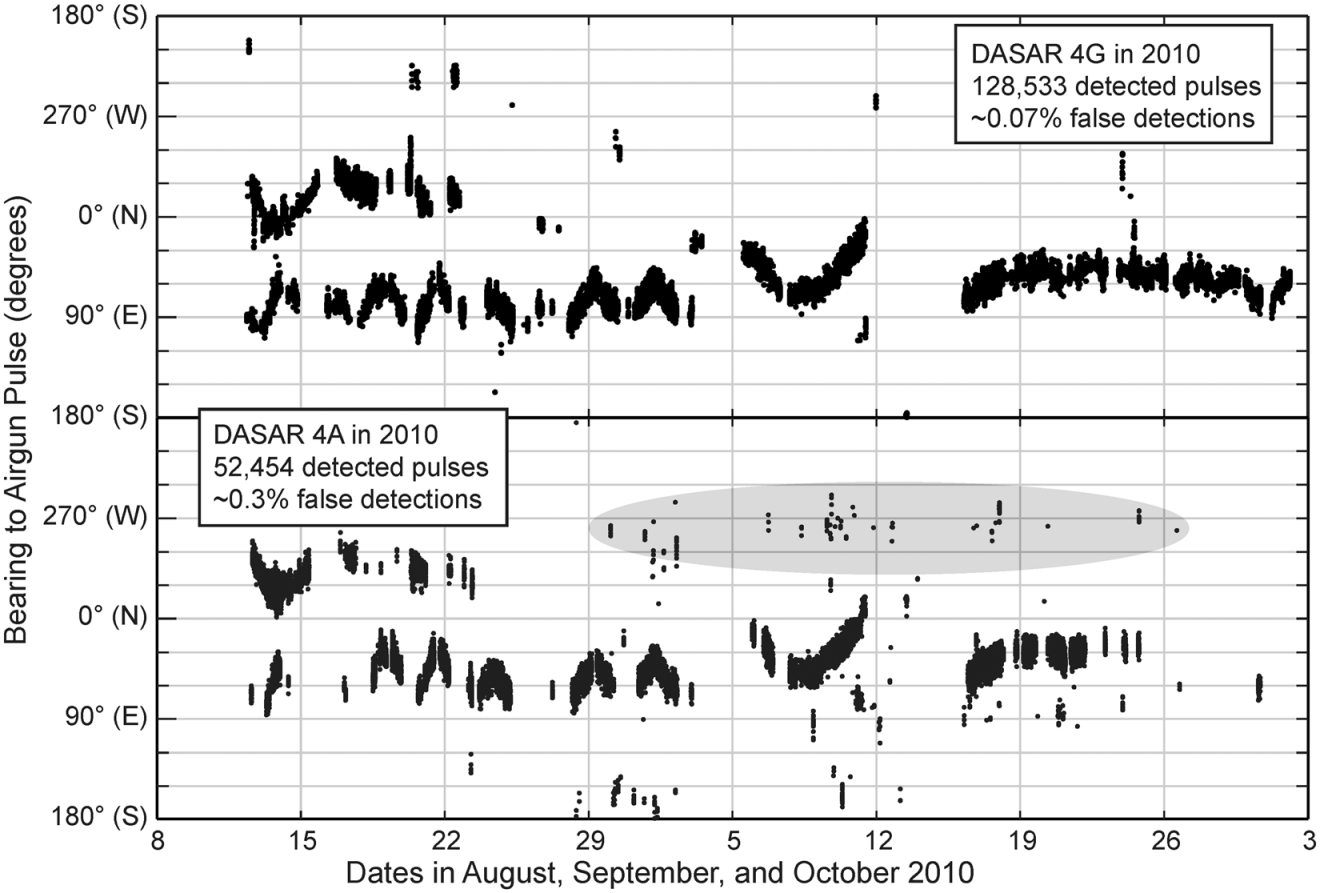
Bearings to airgun pulses as detected at DASARs 4A and 4G during the 2010 season. The originators of these airgun pulses were two distant operations (see section *Airgun Activities during 2007–2010* above). The gray oval highlights examples of false detections (i.e., impulses of sound that are not airgun pulses). In deeper water (i.e., DASAR 4G) the number of detected pulses is higher and the percentage of false detections is lower. See text for more information.

Fig 4 shows the output from the airgun pulse detector for locations 4A and 4G in 2010, in which bearing is plotted as a function of time. Airgun pulses from two known distant operations (CGS / USGS and GXT, see previous section) can be readily identified. For the CGS / USGS operation, positions of the seismic ship were provided [26], allowing us to confirm the bearings obtained by the airgun pulse detector. Isolated detections in Fig 4, for example in the gray highlighted oval for DASAR 4A, are likely false detections in the detection algorithm and do not correspond with actual airgun pulses. Such false detections were more prevalent in the shallower (southernmost) parts of each array, likely due to the more complex acoustic propagation environment. In addition, at least twice as many airgun pulses were detected by the deeper DASARs at the northern ends of the arrays, because the higher modal propagation cut-off frequencies and relatively larger bottom attenuation at shallower water depths led to lower received levels at the shallowest DASARs. For example, for the shallower DASAR 4A (Fig 4, bottom) ~52,500 airgun pulses were detected in 2010, of which ~0.3% were deemed to be false detections. Concurrently, >128,500 airgun pulses were detected at the deeper DASAR 4G with ~0.07% of isolated (noise) detections. Outputs from the airgun pulse detector, such as the ones shown in Fig 4, were examined for each DASAR, each year, and compared to the locations of known seismic operations. At site 1 in 2007 and 2010 the percentage of non-airgun pulse false detections was much higher than the percentage of false detections shown in Fig 4. Site 1 was the shallowest site, with the fewest detected airgun pulses. Because of the shallow water, only nearby operations were detected consistently. Since we had detailed position information for the nearby operations that took place in 2007 and 2010 (see Table 2), we filtered the site 1 data in those two years by removing detections that did not occur at times when airgun arrays were firing at known operations or that did not originate from the general direction (±5° of calculated bearing) of these operations. All other sites yielded consistently high-quality airgun detection estimates.

For each 5, 10, and 20-min period at each functional DASAR in each of the four years, a cumulative sound exposure level (*CSEL*_*t*_) was calculated by summing the sound exposure levels (SELs) of all the airgun pulses detected during the time interval of interest. *CSEL*_*t*_, where *t* is 5, 10, or 20 min, in dB re 1 μPa^2^-s, was calculated as follows:

$$CSEL_{t}=10{log}_{10}(\sum_{i=1}^{n}10^{SEL_{i}/10})$$ (1)

where *SEL*_*i*_ represents the *i* ^th^ of *n* pulses detected in the interval *t* [30]. With the exceptions for false detections explained above for site 1 in 2007 and 2010, all pulses detected by the airgun pulse detector were included. If no airgun pulses were detected during a particular period, then a missing value was assigned to *CSEL*_*t*_ (i.e., *CSEL*_*t*_ was undefined for that period, and that cell-time interval was not used in most of the analyses). Note that SEL levels were unweighted in this study. Because of the frequency characteristics of the recorders and of the sound source, M-weighting, which is appropriate for low-frequency cetaceans (as defined in [30]), would not have made a meaningful difference in received SEL values [30, 31].

The cumulative sound exposure metric was chosen in this study because it allowed us to calculate received sound over time as a “dose” that takes into account both the number of pulses received by the whale and the amplitude of those pulses. A single mean or median sound pressure level extracted from a distribution of such measurements over a 10-min period, for example, could yield the same value for a 10-min period containing one pulse or fifty pulses, and is therefore not a good measure of the total (integrated) airgun signal energy to which a bowhead would have been subjected.

The omnidirectional sensor used in DASARs overloads when received levels exceed 151 dB re 1 μPa 0-to-peak (at 100 Hz). When the seismic ship was less than 20–30 km from the DASARs it was not unusual that close to 100% of detected airgun pulses were overloaded. Nevertheless, the computed received levels for overloaded pulses still provide an important piece of information in the framework of this study: they represent a minimum level for each received airgun pulse. (Because overloading is defined based on a peak pressure value, SELs for overloaded pulses varied by >20 dB.) In addition, based on previous investigations [19] we expected the thresholds for behavioral change to be well below the levels at which the DASARs overloaded. Therefore, instead of dismissing these pulses, they were flagged in the records. When *CSEL*_*t*_ was calculated for each time interval, the percentage of the pulses that were overloaded was also computed.

### Call Response Parameterization and Poisson Regression Model

The fundamental goal of this analysis was to determine the relationship between the received level (RL) of sound from airgun pulses (as measured by *CSEL*_*t*_, see above) and whale calling rates. To this end, two statistical analyses were conducted. The most straightforward was a set of simple t-test comparisons to determine whether the mean calling rate during times with and without airgun pulses were statistically different. This approach also gave a first order perspective on the type of model for which to aim. A more sophisticated approach involved fitting a non-linear Poisson regression model to our call rate vs. RL dataset, while confidence intervals for model parameters were estimated via block bootstrapping. Because the t-tests rely on concepts introduced in the Poisson regression modeling, they will be presented after the modeling in the section ***Comparing Plateau and No-Seismic Calling Rates***.

**Fig 5:**
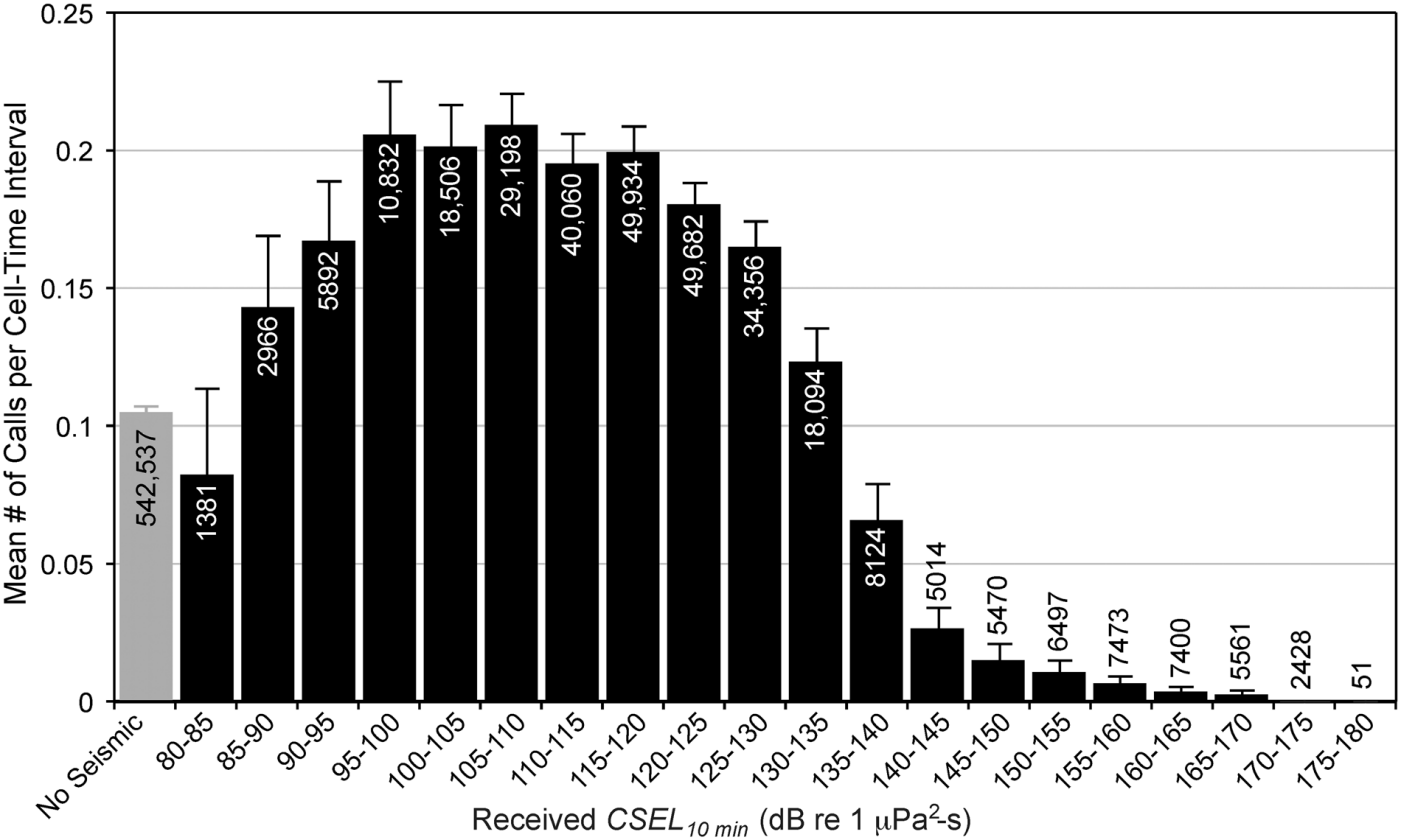
Calls per cell-time interval as a function of received level of sound from airgun pulses. Mean number of localized bowhead calls per cell-time interval for 20 5-dB bins of received *CSEL*_*10-min*_, as well as a *no-seismic* category (gray bar) containing all the cell-time intervals without any detected airgun pulses. Upper 95% confidence limits are shown on each bar. Data from all four years and all five sites are included. Sample sizes, i.e., the number of cell-time intervals in each bin, are shown inside or above the bars. Note that this plot excludes 888 cell-time intervals with received *CSEL*_*10-min*_ below 80 dB re 1 μPa^2^-s (see text).

**Fig 6:**
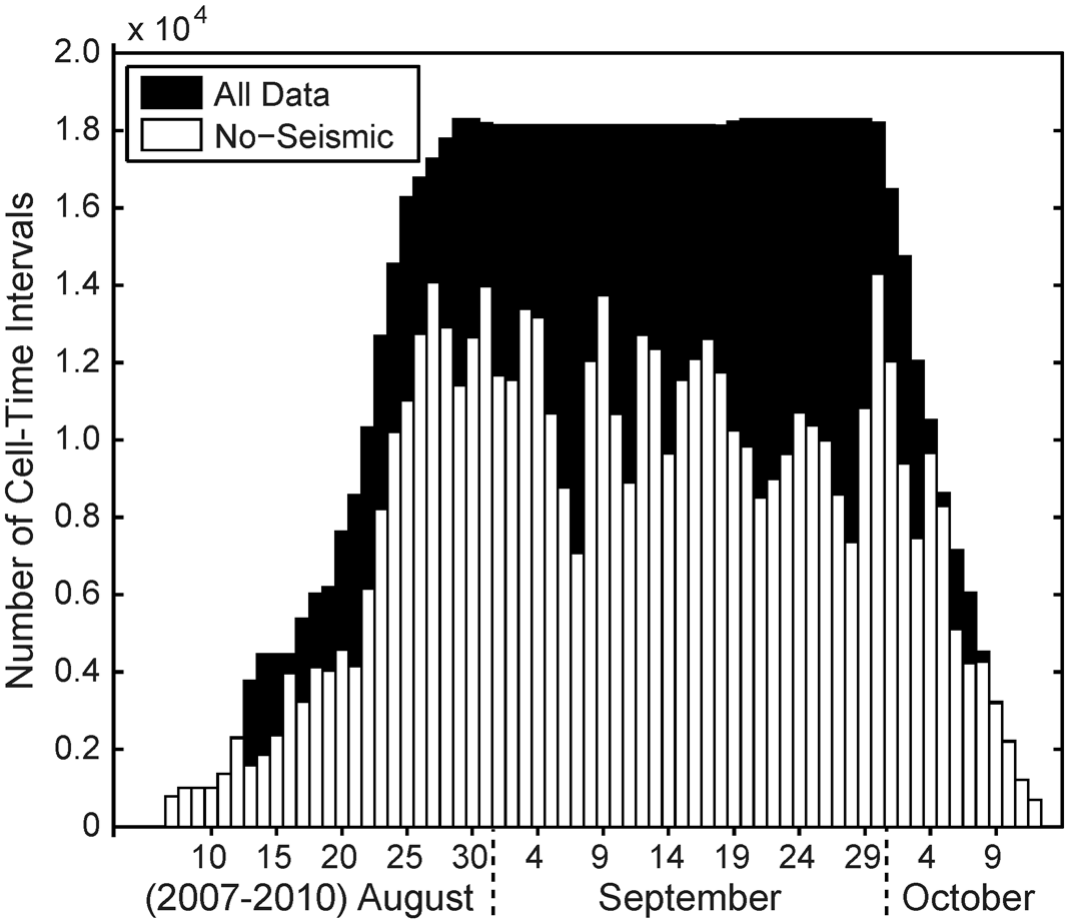
Frequency distribution of samples without detected airgun pulses, as a function of date. The black bars show the total number of cell-time intervals while the white bars show the number of cell-time intervals without any detected airgun pulses, as a function of date in all four years (2007–2010). The top, nearly flat part of the black bars corresponds to the dates during which all DASAR arrays were deployed in all years. The right edge of the plot is steeper than the left edge because the period of retrieval in October was less variable in timing than the period of deployment in August (see Table 1). The figure shows that cell-time intervals without detected seismic activity are not biased toward certain phases of the migration, and thus any differences between calling rates found between *no seismic* and *seismic* cell-time intervals are not contaminated by seasonal effects.

To get an idea of how to model call production rate in terms of airgun CSEL_*t*_, the mean calling rate per 10-min period was plotted as a function of received *CSEL*_*10-min*_, for the entire dataset, including 10-min periods during which no airgun pulses were detected (Fig 5). (As discussed below, a sensitivity analysis of the 5, 10, and 20-min CSEL integration times found that 10 min was an appropriate integration interval for the analysis that follows.) Fig 5 shows that mean calling rate for times with no detected airgun pulses (“*no-seismic*” category) was about 0.1 calls per cell-time interval. Note that this category has about twice as many samples as all other CSEL categories combined. To ensure that the *no-seismic* calling rate was computed from samples spread over the entire season, the number of *no-seismic* cell-time intervals was tallied every day in all four years and compared to the total number of cell-time intervals (Fig 6). This plot confirms that *no-seismic* cell-time intervals are not biased to certain phases of the migration season. For example, there are fewer calls in mid- to late-August when the fall migration begins, but a peak occurs in call detection rates in mid- to late September. Thus, one needs to confirm that the *no-seismic* category samples all time periods throughout the seasons. If it did not, one could argue that any differences seen between *no-seismic* call rates and other cell-time categories arise from seasonal differences in bowhead whale sound production rates, and not changes in behavior. Fig 6 shows that *no-seismic* samples occur during all parts of the season.

Fig 5 shows a surprising result: as received *CSEL*_*10-min*_ increased from barely detectable to high amplitude airgun pulses, calling rates initially *increased*, then stabilized and peaked, and then *decreased* abruptly towards 0 as received *CSEL*_*10-min*_ increased further. The magnitude of these responses exceeded the 95% confidence limits and were thereby judged to be a real effect. Therefore, we sought a model to capture this fundamental plateau structure in call responses. Here, we refer to the levels at the transitions on either side of the plateau as “thresholds”. We define the lower threshold (Δ_1_) as the point at which calling rates reach the plateau, and the higher threshold (Δ_2_) as the point at which calling rates begin to drop away from the plateau.

The regression model estimated the two thresholds and their variability, with the response variable defined as the number of calls located within a particular analysis cell during a particular time interval *t* (of duration 5, 10, or 20 min). However, this response variable also depends on factors other than accumulated sound from airgun pulses, such as water depth. Therefore, it was necessary to estimate the thresholds while simultaneously accounting for these other effects. Preliminary analyses showed that most of these other factors are correlated with the DASAR site. For example, the mean depth of DASARs at a site is strongly associated with site number. These factors could therefore be incorporated into the regression model by simply including site number as a categorical factor in the analysis.

We chose to disregard any cell-time intervals with a *CSEL*_*t*_ value below 80 dB re 1 μPa^2^-s. Blackwell *et al*. [32] computed whole-season minimum percentile background levels for two DASARs at each site (generally locations A and G) in each of the four years 2007–2010. The median level of the 5^th^ percentile at all 40 DASARs (5 sites x 2 DASAR locations x 4 years) was ~80 dB re 1 μPa (rms), with ~60% of the median levels between 75 and 85 dB re 1 μPa. Therefore, a cell-time interval with a *CSEL*_*t*_ below 80 dB could only occur at the quietest times of the season (less than 5% of the time) with a few barely detectable airgun pulses. For example, two airgun pulses with received SELs of 78 dB re 1 μPa^2^-s yield a *CSEL* of 81 dB re 1 μPa^2^-s, which exceeds the 80 dB cut-off.

Estimates of the thresholds were derived from a non-linear Poisson regression model relating the number of calls in a particular cell-time interval to *site* (represented as a categorical variable) and *CSEL*_*t*_ (represented by two threshold functions). Poisson regressions are a specific class of generalized linear models, a well-established branch of statistical regression theory. The non-linear Poisson regression took the form

ln(E[yij])=β0+β1s1ij+β2s2ij+β3s4ij+β4s5ij+β5H1(xij,Δ1)(Δ1-xij)+β6H2(xij,Δ2)(xij-Δ2) (2)

where *y*_*ij*_ is the number of calls located in cell *i* during time interval *j*, *E*[*y*_*ij*_] denotes the expected value (or average) of *y*_*ij*_, *s*_*kij*_ are indicator functions for observations from site *k* during interval *j* at cell *i* (i.e., *s*_*kij*_ = 1 if *y*_*ij*_ is a count from an analysis cell at site *k*, *s*_*kij*_ = 0 otherwise). Note that indicator functions (*s*_*kij*_) for site 3 were absent because it was chosen as the reference site; i.e., the intercept, *β*_0_, represented the site 3 effect and the coefficients *β*_1_ to *β*_4_ represented the differential effects of the other four sites relative to site 3. *x*_*ij*_ is the *CSEL*_*t*_ for analysis cell *i* during interval *j*, *H*_*a*_(*x*_*ij*_, Δ_*a*_) is a logistic approximation to the Heaviside step function defined below [33, 34], and the Greek symbols *β*_0_, …, *β*_6,_ Δ_1_, and Δ_2_ are all parameters to be estimated. The logistic approximations *H*_*a*_(*x*_*ij*_, Δ_*a*_) are parameterized as

$$H_{1}(x_{ij},\Delta_{1})=\frac{1}{1+exp[-\kappa_{1}(\Delta_{1}-x_{ij})]}$$ (3)

and

$$H_{2}(x_{ij},\Delta_{2})=\frac{1}{1+exp[-\kappa_{2}(x_{ij}-\Delta_{2})]}$$ (4)

In Eq (2), *β*_5_ and *β*_6_ are the slopes towards and from the two thresholds, and Δ_1_ and Δ_2_ are the values of the thresholds in *CSEL*_*t*_. In Eqs (3) and (4), *κ*_1_ and *κ*_2_ represent the “knees” which control the rate of change in the immediate vicinity of the thresholds; these parameters were not estimated but rather were fixed as they had no discernible effect on the fundamental shape of the function nor on estimation of the other parameters. Our primary interests were in the threshold parameters Δ_1_ and Δ_2_, the former being the threshold value at which calling rates reach the plateau, and the latter being the threshold value at which calling rates begin to decrease.

We conducted bootstrapping to estimate parameter variances without making explicit distributional assumptions, while also accounting for potential serial correlation in whale call counts. Ninety-five percent confidence intervals for the regression model parameters were computed by block bootstrapping [35]. For more details about these methods, see S4 File.

### Comparing *Plateau* and *No-Seismic* Calling Rates

A simple test was used to inquire whether there was a significant difference in calling rates between *no-seismic* periods (leftmost bar in Fig 5) and the *plateau* region of the distribution (the area between the two estimates for the thresholds Δ_1_ and Δ_2_ using *CSEL*_*10-min*_). Mean calling rates (calls / 10 min) were calculated for each site separately for the *no-seismic* and *plateau* periods. This reduced all observations (851,456 cell-time intervals, i.e., the sum of all the samples shown in Fig 5) to 10 mean values, two for each of the five sites. The site effect is substantial, that is, the calling rate at the five sites varies due to natural factors, such as the location and spread of the migration corridor or water depth. Therefore, it makes sense to treat the site as a blocking factor and since there are two observations per site, a natural test for the difference in calling rates is the paired *t*-test. This test is a one-sample test for whether the mean difference (i.e., the mean of the differences between *no-seismic* and *plateau* mean calling rates) differs from 0. However, rather than the absolute differences, relative differences were tested because overall calling rates are quite different between sites. The relative difference at the *i* ^th^ site was calculated as

$$d_{i}=\frac{\left(x_{2,i}-x_{1,i}\right)}{x_{1,i}}$$ (5)

where *x*_2,*i*_ was the mean *plateau* calling rate (between the two thresholds) and *x*_1,*i*_ was the mean *no-seismic* calling rate. Having prior knowledge that the *plateau / seismic* rate exceeds the *no-seismic* rate, a one-sided test was performed to test whether the *plateau / seismic* rate is indeed significantly greater than the *no-seismic* rate.

The test statistic is

$$t=\frac{{\overset{-}{x}}_{d}}{s_{d}/\sqrt{5}}$$ (6)

where ${\overset{-}{x}}_{d}$ is the mean difference, i.e., ${\overset{-}{x}}_{d}=\sum_{i=1}^{5}d_{i}/5$, and *s*_*d*_ is the standard deviation of the differences. These paired *t*-tests were conducted on three data sets, all using the 10-min time intervals: (a) all four years combined; (b) 2007 and 2008 combined, the two years with a lot of nearby (< 50 km distant) airgun activity; and (c) 2009 and 2010 combined, the two years with primarily distant airgun activity. The division of the data in cases (b) and (c) was performed to see whether the initial increase in calling rates over *no-seismic* rates observed in Fig 5 was affected by the range of the airgun survey activities.

## Results

### Whale Call and Airgun Pulse Counts

Over the course of the study, 975,657 bowhead whale calls were localized at the five sites, as shown in section A of Table 3. Of these, 106,324 (~11%) were located inside the 42 analysis cells (Table 3, section B). The number of calls that were used in model estimation was 49,297, or about 5% of the total number of localized calls (Table 3, section C). This smaller number includes only calls from cell-time intervals with concurrent airgun pulse detections.

**Table 3:** Numbers of localized whale calls.

Year	Site 1	Site 2	Site 3	Site 4	Site 5	Sums
A. Total number of localized calls						
2007–2010	49,890	134,111	214,724	275,496	301,436	**975,657**
B. Number of localized whale calls inside analysis cells						
2007	1997	4842	4485	4111	6870	22,305
2008	3354	14,375	7655	7226	9908	42,518
2009	819	1547	1418	1713	6760	12,257
2010	1053	0	6240	14,777	7174	29,244
Sums	7223	20,764	19,798	27,827	30,712	**106,324**
C. Number of localized calls that occurred while airgun pulses were concur-rently being detected						
2007	42	1186	1118	833	3218	6397
2008	814	8174	3939	3098	8359	24,384
2009	42	558	798	772	3561	5731
2010	1	N.A.	2197	6012	4575	12,785
Sums	899	9918	8052	10,715	19,713	**49,297**

Section A includes all calls localized, regardless of their distance from the DASAR arrays. Section B includes all calls localized inside the 42 analysis cells. Section C shows the number of calls localized inside the analysis cells during 10-min time intervals with concurrent airgun pulse detections. These calls were used in the model fitting.

**Fig 7:**
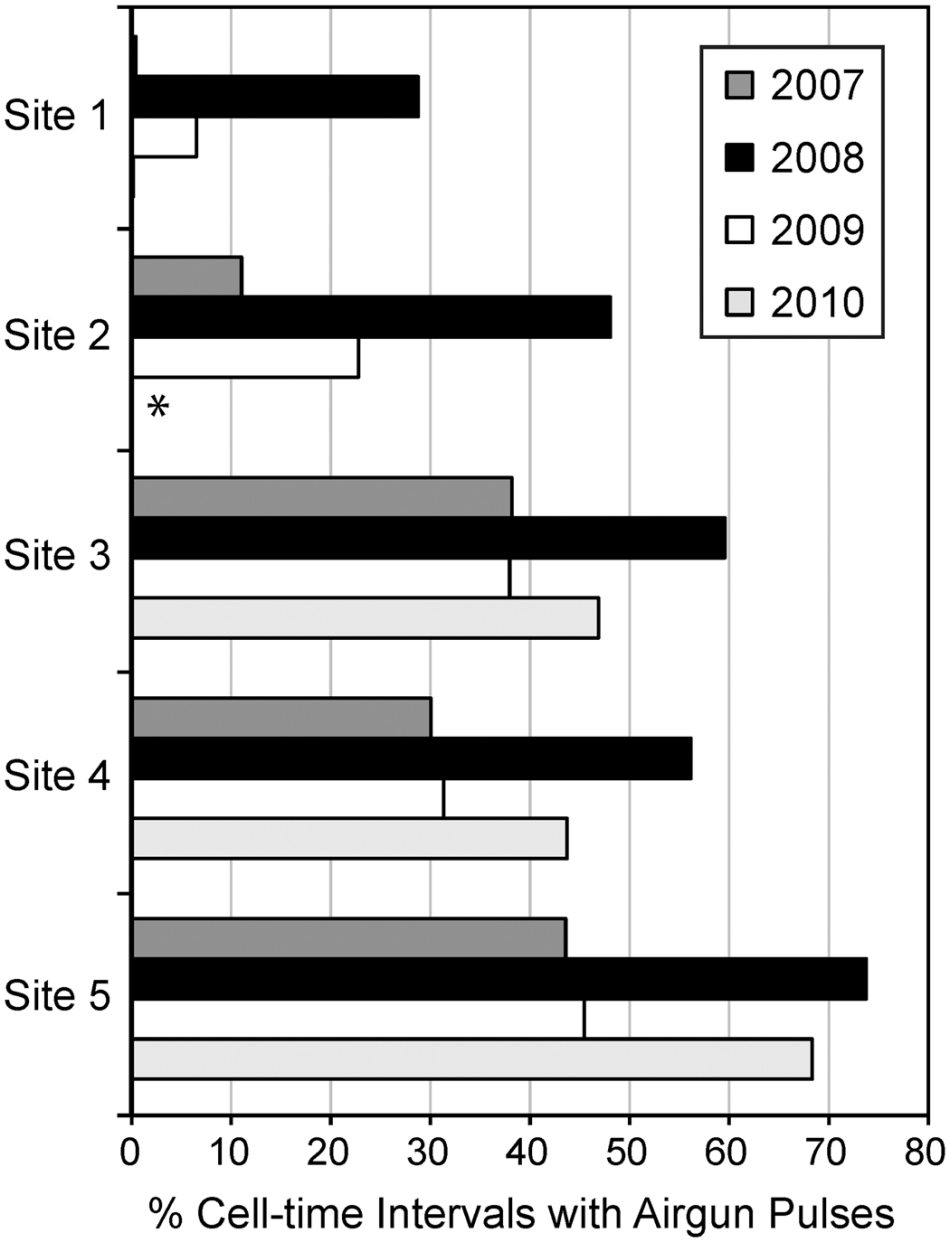
Percentage of 10-min cell-time intervals with airgun pulse detections, as a function of site and year. There are no data for site 2 in 2010 (*) as most of that site was not deployed due to ice.

Over the course of the study a minimum of ~628,000 separate airgun pulses were detected, representing nearly 11 million detections at all DASARs combined. A summary of the numbers of airgun pulses detected and statistics of the derived pulse parameters are shown in S2 Table. The percentage of cell-time intervals with detectable airgun pulses at each site was quite variable and is shown in Fig 7 (using 10-min intervals). This percentage evidently depended on the location of the airgun array(s), but also on DASAR deployment depth. Since shallow waveguides heavily attenuate low-frequency signals, site 1—the shallowest site—always detected the fewest airgun pulses (as well as the fewest whale calls). The year 2008 had the most seismic exploration within the area of the DASAR arrays and not surprisingly the highest percentages of cell-time intervals with detected airgun pulses (Fig 7). Site 5 was the farthest away from Shell’s “nearby” airgun operations in 2007, 2008, and 2010, but its location in the deepest water (on average 48 m) made it well-suited for detecting distant airgun pulses, including those from the GSC / USGS operation to the north and operations to the east in the Canadian Beaufort. In 2009 and 2010, the nearest seismic exploration was hundreds of km from site 5, yet 45% and 68%, respectively, of 10-min intervals at that site contained airgun pulses from distant operators (Fig 7).

### Estimating the Thresholds

The lower threshold Δ_1_, at which bowhead whale calling rates reach a plateau, was estimated at 92.0 dB, 94.5 dB, and 97.1 dB re 1 μPa^2^-s for time intervals of 5 min, 10 min, and 20 min, respectively (Table 4). For each doubling of the time interval the point estimate increased by ~2.5 dB. The 95% confidence intervals were ~35 dB, 13 dB, and 17 dB for 5, 10, and 20-min periods, respectively. The fitted Poisson regression models are summarized in Table 4 (thresholds and corresponding confidence intervals) and S3 Table (all parameters) for the three time intervals.

**Table 4:** Poisson regression model parameter point estimates and bootstrap confidence limits for three alternative time intervals.

Time interval	Parameter	Interpretation	Pt estimate	L95	U95
**5 min**	Δ_1_	Lower threshold	92.0	85.8	120.7
	Δ_2_	Upper threshold	124.6	120.1	127.3
**10 min**	Δ_1_	Lower threshold	94.5	87.6	100.8
	Δ_2_	Upper threshold	127.4	122.9	129.9
**20 min**	Δ_1_	Lower threshold	97.1	87.8	105.2
	Δ_2_	Upper threshold	130.5	125.7	132.4

L95 and U95 represent the lower and upper 95% confidence limits, respectively.

The upper threshold Δ_2_ at which calling rates begin to decline was estimated at 124.6 dB, 127.4 dB, and 130.5 dB re 1 μPa^2^-s for time intervals of 5 min, 10 min, and 20 min, respectively (Table 4). As expected, and similarly to the lower threshold, each doubling in the length of the time interval led to an increase of ~3 dB in the *CSEL*_*t*_ value. For each of the three time intervals, the 95% confidence intervals spanned ~7 dB and overlapped each other; they were therefore smaller and less variable than those for the lower threshold. Predicted calling rates were near 0 (less than 0.02 calls per cell-time interval) when *CSEL*_*t*_ levels exceeded 160 dB. Between the two thresholds, a range of received CSELs of about 33 dB re 1 μPa^2^-s for all three time intervals (Table 4), calling rates remained high.

**Fig 8:**
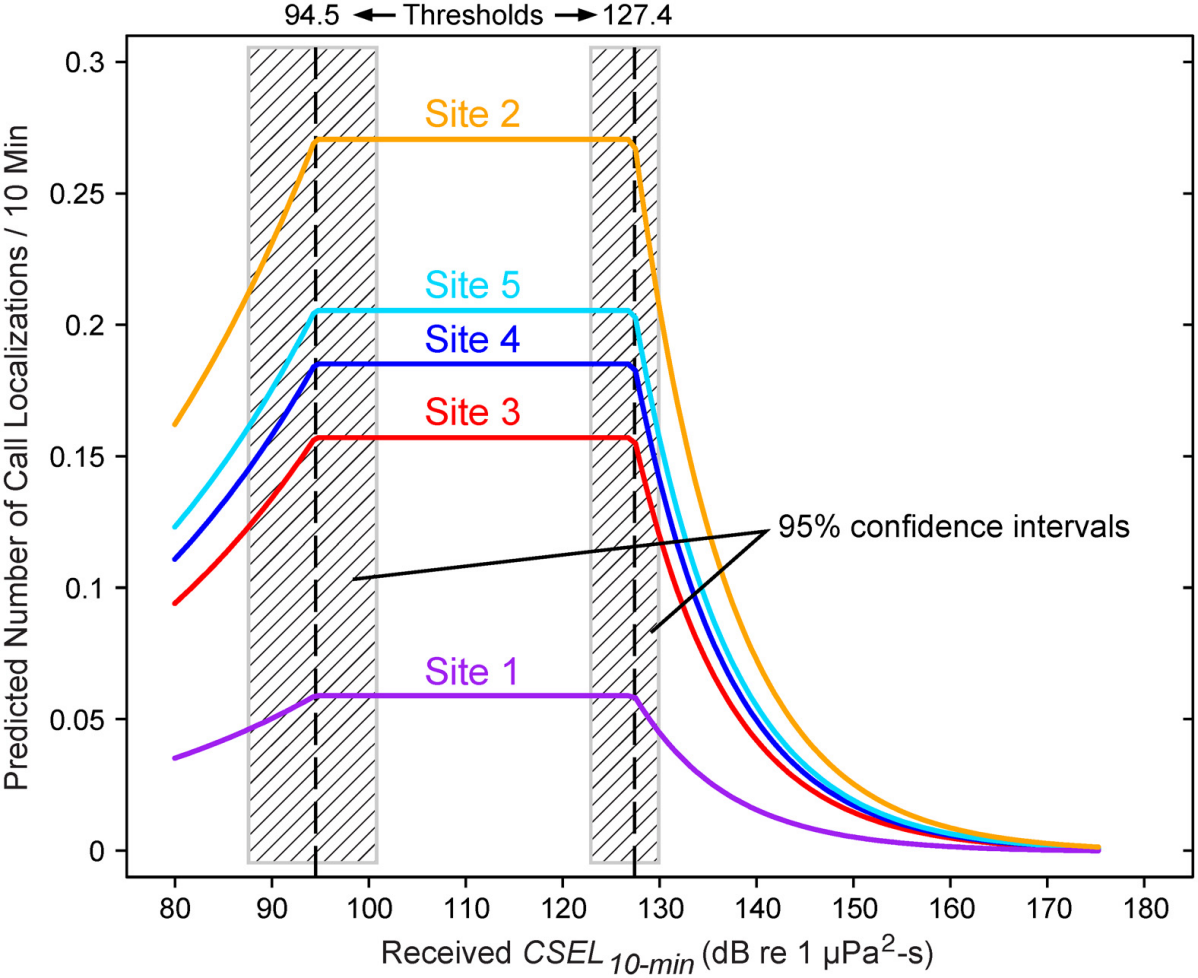
Threshold estimates from Poisson regression model, for all sites and all years combined. The model uses a cumulative sound exposure level calculated over 10-min periods (*CSEL*_*10-min*_). The threshold estimates are 94.5 and 127.4 dB re 1 μPa^2^-s, respectively. Hatched areas are the 95% confidence intervals. Different sites had different predicted call localization rates per 10-min interval. See text for more information.

In a more detailed look at the results, presented below, we focus on the analysis done with 10-min intervals. Data from all years and all sites were combined in these analyses, but there was a site effect in that the predicted mean number of calls per cell-time interval at the plateau differed between sites. This is demonstrated in Fig 8, which shows the final model, the threshold estimates, and the 95% confidence intervals for the 10-min time interval. Fig 8 also shows that for cell-time intervals with detected airgun pulses, site 2 had the highest calling rate, followed by site 5, site 4, site 3, and site 1. On average, the peak calling rate at site 2 was 5–6 times that at site 1.

**Fig 9:**
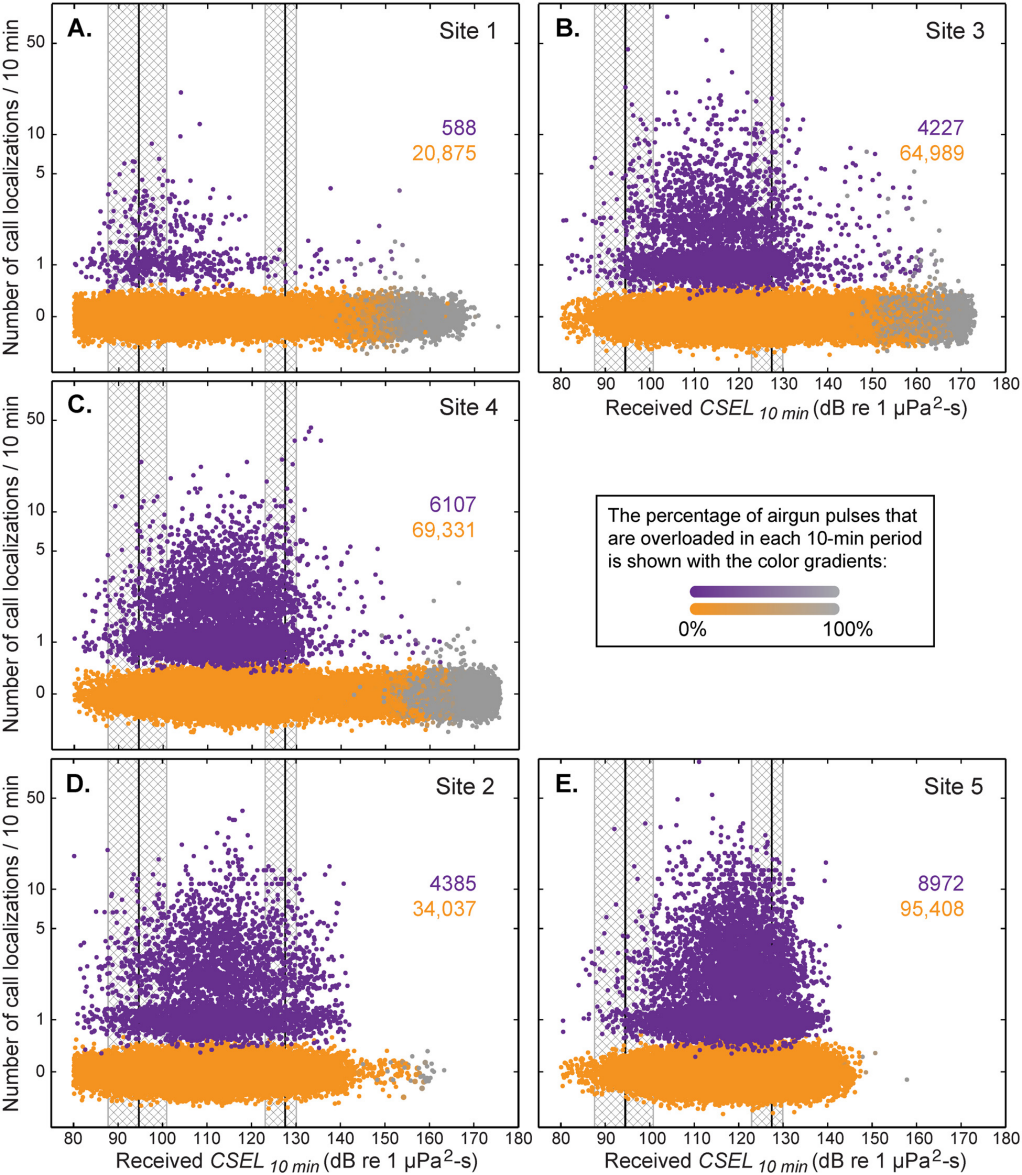
Relative effects of received CSEL_*10-min*_ on call localization rate, for all five sites. (A), (B), and (C) (sites 1, 3, and 4, respectively) show the data for sites with nearby airgun use, while (D) and (E) (sites 2 and 5, respectively) show the data for sites that only received relatively distant airgun pulses (see text). All four years of data are combined. Each point represents a cell-time interval, and shows the number of localized calls detected during that time window as a function of the received CSEL_*10-min*_. The two thresholds are shown with black vertical lines and the crosshatched areas are the 95% confidence intervals. The orange and purple numbers in each plot are the numbers of cell-time intervals with zero versus one or more localized calls per 10-min period, respectively. See text for more information.

Fig 9 shows the actual data sets used for the modeling, displayed by site (all years combined), for the 10-min time interval. Sites 1, 3, and 4 were close to the seismic operations by Shell in 2007, 2008, and 2010, and are shown in the top 3 plots [***(A)***, ***(B)***, and ***(C)***]. Sites 2 and 5 were tens to hundreds of km away from these operations and are shown in the bottom 2 plots [***(D)*** and ***(E)***]. Each dot on these plots represents an analysis cell at a particular 10-min time window in the fitting data set (i.e., a cell-time interval), and is shown as a function of the received *CSEL*_*10-min*_ and the number of localized calls detected during that time window. For purposes of display only, vertical coordinates of points from 0 to 9 have been “jittered” to show overlapping points. Without jittering, all points with the same number of calls would fall on a horizontal line. Dots are colored according to the number of localized whale calls and the percentage of airgun pulses that were overloaded in each 10-min time window: cell-time intervals with no localized whale calls and no overloaded pulses are shown in orange, whereas cell-time intervals with at least one localized whale call and no overloaded pulses are shown in purple. With an increasing percentage of overloaded pulses (per 10-min interval), both colors transition gradually to gray. Not surprisingly, sites 2 and 5, farthest away from the seismic exploration, had very few cell-time intervals with overloaded pulses (actual percentages for overloading are given in S2 Table). The numbers of cell-time intervals with 0 (orange) versus 1 or more (purple) call localizations are also given in each plot. For site 1, there were 35.5 times more cell-time intervals without calls than with calls (20,875 / 588 = 35.5). This ratio was intermediate for sites 3 and 4 (15.4 and 11.4) and lowest for sites 2 and 5 (7.8 and 10.6).

### Comparing *Plateau* and *No-Seismic* Calling Rates

Results from the *t*-test comparison of calling rates between the *plateau / seismic* and times with no detected airgun pulses (*no-seismic* calling rate) are shown in Table 5. In the analysis for all years combined and 2007–2008 (sections A and B in Table 5), the *plateau* calling rates were consistently greater than the *no-seismic* calling rates at all five sites and therefore the differences all have the same sign. Both comparisons were statistically significant (*P*<0.05), showing that the whales’ heightened calling rate in the presence of low levels of received airgun pulses was statistically different from calling rates in the absence of seismic operations. In contrast, when the comparison was limited to 2009 and 2010 (section C in Table 5), the two years with distant seismic operations or low-level shallow hazard surveys using a single airgun (see Table 2), the increase in calling was non-significant (*P* = 0.183).

**Table 5:** Calling rate differences with and without the presence of airgun pulses.

		Mean calling rate				
	Site	No seismic	Plateau / seismic	Relative difference		
**A. All years**	1	0.0377	0.0608	0.6146	n:	758,582
	2	0.1091	0.2624	1.4043	Mean:	0.5673
	3	0.1412	0.1579	0.1178	S.D.:	0.5059
	4	0.1475	0.1828	0.2395	t-stat:	2.5076
	5	0.1444	0.2109	0.4605	p-value:	0.033
**B. 2007–2008**	1	0.0534	0.0648	0.2126	n:	388,233
	2	0.1413	0.3218	1.2776	Mean:	0.6821
	3	0.1619	0.2267	0.3996	S.D.:	0.4087
	4	0.1531	0.2727	0.7816	t-stat:	3.7325
	5	0.1409	0.2451	0.7393	p-value:	0.0102
**C. 2009–2010**	1	0.0219	0.0197	-0.0967	n:	370,349
	2	0.0334	0.0638	0.9097	Mean:	0.1917
	3	0.1182	0.1052	-0.1104	S.D.:	0.42
	4	0.1435	0.1531	0.0667	t-stat:	1.0208
	5	0.1477	0.1757	0.1892	p-value:	0.1826

Mean calling rate (calls per 10-min cell-time interval) is shown for “no-seismic” and “seismic / plateau” periods, at all sites, for three different data sets: (A) combining all years, (B) the two years (2007–2008) with seismic exploration near the DASAR sites, and (C) the two years (2009–2010) with only distant seismic exploration. The relative difference is defined in Eq (5). The sample size (n) is the number of cell-time intervals used in each category.

### CSEL Thresholds Relative to Distances from the Seismic Ship

It is useful to put these CSEL thresholds in context: For example, at what distance from an airgun array will the upper threshold of 127.4 dB re 1 μPa^2^-s be reached, leading whales to start calling less?

The *M/V Gilavar* was the seismic ship involved in Shell’s seismic exploration in 2007 and 2008 (see Fig 3 and Table 2). This vessel used a 24-airgun, 3147 in^3^ array during full operations, a single 30 in^3^ mitigation airgun, and triggered the airguns every 10 s. In both 2007 and 2008, sound source characterizations (SSCs) were performed on the *Gilavar’s* airgun arrays [36, 31]. Both SSCs were performed in our study area, near sites 3 and 4 in 2007, and near site 1 in 2008. The empirical data collected were used to estimate the distances from the seismic ship at which various behavioral changes in whale calling behavior are predicted to take place.

If we assume 60 airgun pulses per 10-min period and, for the sake of simplicity, a constant received pulse SEL, the resulting *CSEL*_*10-min*_ value is 17.8 dB above the pulse SEL value, e.g., for a pulse SEL of 100 dB: 10 log_10_(60(10^100 / 10^)) = 117.8 dB re 1 μPa^2^-s. In other words, a *CSEL*_*10-min*_ value of ~127.4 dB re 1 μPa^2^-s—the upper threshold, when calling rates start to drop—corresponds to a single-pulse SEL of 109.6 dB re 1 μPa^2^-s. (Note that this is a simplified example. A *CSEL*_*10-min*_ level of 127.4 dB could also be achieved with fewer pulses at higher SELs or more pulses at lower SELs). Table 6 shows that a received level of ~110 dB SEL was reached about 50 km from the *Gilavar* using its full array based on the 2008 SSC [31]. The other *CSEL*_*10-min*_ value shown in Table 6 is 160 dB re 1 μPa^2^-s, when very few calls were detected in our analysis (see Fig 9). Based on the 2008 SSC, the predicted distance at which this would occur was 10–20 km from the seismic ship (Table 6).

**Table 6:** Distances from the *M/V Gilavar* at which various threshold levels are reached. This table uses empirical data collected during sound source characterizations (SSCs) in the same study area in 2007 and 2008 [36, 31]. Note that regressions for sound exposure levels were not included in the reports, so the distances in this table are estimated visually from data plotted in the listed figures. CSEL = cumulative sound exposure level, SEL = sound exposure level.

	*CSEL*_*10-min*_	Single-pulse SEL	2007 SSC		2008 SSC	
	*dB re 1 μPa* ^*2*^ *-s*		Full array^a^	Mitigation airgun^b^	Full array^c^	Mitigation airgun^d^
**Upper threshold**	127.4 dB	~110 dB	~100 km^e^	~40 km^e^	~50 km	20–30^e^ km
**Almost complete lack of calls**	160 dB	~142 dB	30–40 km	~6 km	10–20 km	~2 km

^a^ See Fig 3.19 in [36]

^b^ See Fig 3.21 in [36]

^c^ See Fig 55 in [31]

^d^ See Fig 57 in [31]

^e^ These distances were visually interpolated or extrapolated from the figures listed above. They are very coarse and only intended to provide a rough-order estimate of distances.

The lower threshold (94.5 dB)—when calling rates in the presence of airgun pulses reached the plateau (highest) level—is not included in the Table 6. Cell-time intervals with *CSEL*_*10-min*_ values below 94.5 dB generally included only a few airgun pulses (3 or fewer pulses for ~30%, 6 or fewer pulses for ~50% of cell-time intervals) because they occurred far from seismic operations. The lower threshold is therefore not amenable to a calculation as performed for the upper threshold in the example above. Nevertheless, the fact that the lower threshold is reached with so few detected airgun pulses means that bowhead whale calling rates will rapidly double, compared to non-seismic calling rates (see Fig 5), once airgun pulses are detectable.

The examples shown in Table 6, based on four different SSCs (two array configurations in two different years), are only a few of many possible scenarios. The numbers given in Table 6 should, therefore, be used in a general way, as a rough-order estimate of the radius of a circle around a seismic ship where changes in calling behavior are likely to take place. Table 6 shows that when the *Gilavar* used its full array, few or no whale calls would be expected within ~10–40 km of the ship, and whale calling rates would start decreasing 50 km and more from the seismic ship. Based on the data collected during the 2007 SSC, this distance is likely over 80 km but measurements were not made to that range so we can only speculate based on the shape of the fitted data (see Fig 3.19 in [36]). When the *Gilavar* used the mitigation airgun, source levels were much lower so the expected ranges for changes in calling behavior are much smaller: few or no whale calls would likely be detected 2–6 km from the ship, and a decrease in calling rates would begin 20–40 km from the ship.

An alternative way of expressing the upper threshold is as a received dose of airgun sound per minute. A *CSEL*_*1 min*_ exceeding ~118 dB re 1 μPa^2^-s corresponds to a *CSEL*_*5-min*_, *CSEL*_*10-min*_, or *CSEL*_*20-min*_ exceeding the estimated upper threshold for those three time intervals. In other words, if the received *CSEL*_*1 min*_ at the whales is above 118 dB, calling rates will begin decreasing. S5 File provides some insight into how these CSEL thresholds translate into SPLs, a more commonly used metric.

## Discussion

This analysis has shown two measurable behavioral thresholds in bowhead whales in response to sounds from airgun pulses. At first, as soon as airgun pulses were detectable above ambient levels, bowhead whale calling rates increased over *no-seismic* calling rates. Calling rates increased with received cumulative sound exposure level (CSEL, in units dB re 1 μPa^2^-s and summed over 10 min) until they were about twice the *no-seismic* rate. Calling rates remained high over a ~33 dB range of received CSELs (Fig 8). In addition, at a received CSEL of ~127 dB (equivalently expressed as ~118 dB re 1 μPa^2^-s, as summed over 1 minute), calling rates began to decrease, and were near zero at received CSELs of about 160 dB.

The use of alternative time intervals of 5 min and 20 min did not change these results, other than by shifting the thresholds by ~3 dB, as one would expect with a doubling or halving of the integration time in the calculation of a cumulative sound exposure level. This shows that the results are generally not sensitive to the choice of the time interval, although the variability of the lower threshold increases substantially when the shortest time interval of 5 min is employed. Note that our analysis required an integration time (i.e., 5, 10, or 20 min) but from the perspective of the whale those times are not of any particular importance—they are only a way for us to quantify the dose of airgun sound received by the animal. In most situations, this dose tends to be fairly constant over time, because airgun arrays are normally used for many hours while the seismic ship—and the whales—move relatively slowly. The exception is close to the seismic ship, where received levels will change rapidly over time.

Masking is always a potential concern in passive acoustic studies. It is particularly important that the anthropogenic sound being studied (in this case, airgun pulses) not mask the response of interest (in this case, whale calls) since it would then appear as though animals stop calling when, in fact, calls merely could no longer be detected. Guerra *et al*. [37] showed that reverberation from seismic surveys can substantially increase background levels, particularly within a few km of the seismic ship (e.g., 10–25 dB re 1 μPa within 15 km of a 3147 in^3^ array). In this study, masking of whale calls by airgun pulse reverberation is not believed to be an issue because the received airgun pulse levels at which behavioral changes were detected were low, corresponding to distances from the seismic vessel of tens of km. In addition, by restricting the analysis area for call detection to 2-km circles around each DASAR, whale calls used in the analyses generally had high SNRs (signal-to-noise ratios). At the distances where the thresholds were detected, the received levels of whale calls were usually higher than the received levels of airgun pulses.

### Comparison of Upper Threshold with Results from Other Whale Species

A review of studies investigating the effects of anthropogenic sounds on the vocal behavior of large whales [8, 9, 13, 14, 38, 39] quickly revealed that comparisons with the present study are difficult to make. These other studies addressed a variety of species, used sound sources with differing acoustic parameters (e.g., amplitude and frequency), and the subject animals likely experienced different contexts (e.g., feeding versus migrating, see [40]). Not surprisingly, the changes noted in calling behavior did not all follow the same trend.

We therefore limit our discussion to the effects of sounds from *airguns* on the vocal behavior of large whales. McDonald *et al*. [41] found that blue whales stopped calling when one or more animals were about 10 km from an airgun array, where estimated received sound levels at the whale(s) were 143 dB re 1 μPa peak-to-peak (10–60 Hz band). This corresponds roughly to a pulse sound pressure level 15–20 dB lower [42], or ~123–128 dB re 1 μPa. The corresponding sound exposure level depends on the pulse length at 10 km in the McDonald *et al*. [41] experiment, which is unknown to us. At a distance of 10 km the pulse length could be close to 1 sec, meaning that the single pulse SEL is also ~123–128 dB re 1 μPa^2^-s. Nevertheless, even if the pulse duration was much lower, say 0.2 sec, the corresponding single pulse SEL (from S5 File, 116–121 dB re 1 μPa^2^-s) would still result in some degree of repressed calling in the bowhead data set. In addition, the narrow bandwidth in the McDonald *et al*. [41] study suggests their estimated level is a minimum. The McDonald *et al*. [41] findings do not, therefore, contradict our own.

For sperm whales foraging in the Gulf of Mexico, Miller *et al*. [43] found a 19% drop in buzz rates (a proxy for foraging attempts) when a seismic ship was operating nearby, but the effect was not significant, likely because of a small sample size. A study by Bowles *et al*. [44] in the southern Indian Ocean suggested that sperm whales may have been silenced by a distant seismic operation. In contrast, in an earlier Gulf of Mexico study [45], no observable avoidance of the whales or changes in vocal patterns during feeding dives were observed when the estimated received (single pulse) sound exposure level at the whales was as high as 124 dB re 1 μPa^2^-s. The seismic ship in the Madsen *et al*. [45] study used a 10-s repetition rate. Thus, 10 min of airgun pulses with a RL of 124 dB SEL would result in a *CSEL*_*10-min*_ value of 141.8 dB re 1 μPa^2^-s, well above our threshold of ~127 dB.

In summary, studies of the effects of airgun pulses on the calling of blue whales and sperm whales have shown either a drop in vocalization rates or no detectable effect. Nevertheless, small sample sizes, dissimilar acoustic units and differing methodologies make comparisons with the present study difficult.

### The Existence of Two Behavioral Thresholds Provides Insight Into Past Bowhead Studies

One of the surprising results of this study is the existence of two behavioral thresholds in bowhead whales’ acoustic responses to seismic activity. To our knowledge, this study is the first to suggest that calling behavior can change in two different ways in response to the same anthropogenic activity, depending on the received levels involved. In retrospect, however, the presence of two thresholds provides insight into some previously perplexing research findings.

In the early stages of this study there was no awareness of the fact that calling rates first increase before they start decreasing—we assumed the “plateau” calling rate was the “normal” calling rate. It was, therefore, puzzling that there seemed to be a positive correlation between the number of airgun pulses detected each year and the number of bowhead calls detected. Even after standardizing across the same dates each year, the highest and lowest whale call counts were obtained in the years with the most and fewest, respectively, airgun pulse detections, with intermediate values for the two intermediate years. Based on the results presented in this paper, this observation can likely be explained by the increased calling which occurs “away” from the general area of the seismic ship, where received levels of airgun pulses result in *CSEL*_*10-min*_ values between the two thresholds. In other words, even though the nearby presence of the seismic ship leads to little or no calling by the whales, the increased calling at more distant sites largely makes up for it.

The existence of a double threshold also sheds new light on previous studies with ambiguous results. For example, Richardson *et al*. [11] showed that bowhead whales exposed to distant seismic pulses exhibited a slight (non-significant) drop in average calling rates. Greene *et al*. [46] found that call detection rates differed significantly at some locations as a function of whether airguns were detectable or not, but the changes were not always consistent. In view of the results presented here, with calling rates first increasing and then dropping to near-zero, a study could readily obtain a non-significant effect if calling rates for whales receiving a wide range of airgun pulse levels were pooled. In other studies, results that could not be explained by the authors at the time now make perfect sense. For example, Greene *et al*. [47] compared bowhead whale call detection rates at several recorders as a function of distance from the airgun arrays. They stated “At the recorder closest to the airguns, call detection rates were lower (P<0.02) at times with pulses than without them. At the recorder farthest [from the airguns], call detection rates were higher when airgun pulses were evident […] than without pulses.”

In an analysis preceding this one, Blackwell *et al*. [19] showed that for bowhead whales relatively near an airgun source (median distance 41–45 km), calling rates dropped when the array became operational. This result is in agreement with the findings of this analysis: At a median distance of ~40 km, RLs at the whales would yield a *CSEL*_*10-min*_ > ~127 dB re 1 μPa^2^-s and calling by the whales would be repressed (see Table 6). The Blackwell *et al*. [19] analysis also showed that for distant whales (median distance >104 km) there was no change in average calling rates when the airguns became operational. Sites 1, 2, and 5 were all lumped into the “far” category in that analysis, but examination of Fig 5 in Blackwell *et al*. [19] shows that when airguns were turned on, calling rates at site 5 fell whereas calling rates at sites 1 and 2 increased. On average, therefore, they did not change, but it is likely that the “far” category included a wide range of CSELs, leading to disparate calling rates. The BACI-type analysis performed in the Blackwell *et al*. [19] study did not have the resolution to detect the subtle shifts in calling rates shown by the present analysis.

### Interpretation of Between-Site Differences

Bowhead whales are long-lived animals [48] that have been exposed to airgun sounds in the Alaskan Beaufort since the late 1960s [49]. There was no expectation of a seasonal habituation or any reason to believe the whales’ reactions should be different at the different sites. Therefore data from all years and all sites were pooled, while retaining a site effect in the model, because preliminary analyses indicated differences in calling rates among the sites (see Eq (2), Fig 8, and paragraph below). *No-seismic* calling rates varied by factors of three to seven between sites. For example, in 2007–2008 the calling rate at sites 3, 4, and 5 (0.14–0.16 calls / cell-time interval) were 2.6–3 times the calling rate at site 1 (0.05 calls / cell-time interval, section C in Table 5). (In 2009–2010 calling rates at site 1 were even lower (0.02 calls / cell-time interval), because immediately following its deployment in 2010, site 1 was covered in dense pack ice for much of the season.) The fact that the migration corridor tends to be wider at site 1 than at sites further east could be a contributing factor, as the stream of whales gets “diluted” when traveling over the westernmost site (see Fig 9.17 in [50]). Nevertheless, the paucity of whales near and west of site 1 has also been noted during aerial surveys conducted by BOEM/NMFS: compared to other coastal survey blocks within our study area, the survey block encompassing site 1 yielded the lowest number of bowhead whales per km surveyed in 2007, 2008, and 2010 [51–53].

The differences in plateau heights among sites (Fig 8) are mainly a result of two factors: the intrinsic between-site differences in calling rates mentioned above, and the distribution of CSELs for each site. For example, site 4 has many more cell-time intervals above the upper threshold (29%) than site 2 (9%), which contributes to the disparate heights of the plateaus for these two sites. Nevertheless, irrespective of site differences, the main findings provided by the model are that calling rates roughly double in response to low levels of airgun pulses and then decrease when the received *CSEL*_*10-min*_ exceeds the upper threshold.

### Possible Effect of Vessel Range on Behavioral Response

Received sound level is not the only factor that influences the behavior of the whale—in this case, the value of the threshold. An animal’s motivational state has been shown to result in gradation of its response to stimuli. Feeding bowhead whales, for example, are less likely to be disturbed by anthropogenic activities than migrating whales [54, 55]. Another factor that could influence behavior, and which is relevant in this study, is the distance between sound source and receiver [40]. There is no reason to assume that a bowhead whale’s reaction to airgun pulses with received SELs of 120 dB re 1 μPa^2^-s will be the same if these pulses come from a large array 100 km from the whale versus a single airgun a few km away. In Fig 9, the purple scatter of points (each representing one or more calls in a cell-time interval) looks somewhat shifted to the right for sites 2 and 5, always distant from the seismic operations, compared to sites 3 and 4, generally much closer to the airguns. Also, the *t*-test analysis found the increase in calling in response to low levels of airgun pulses was significant for the two years with nearby seismic operations (2007–2008, Table 5, section B) but not for the two years with distant or low-level operations (2009–2010, Table 5, section C). Could both of these effects be due to a vessel-range response by the animals, in which the threshold for calling cessation is slightly higher and the increase in calling at low received levels is less pronounced when the whales know the sound source is farther away? Such subtleties in the whales’ responses to sound were beyond the scope of this modeling exercise. Nonetheless, despite being speculative they are worth mentioning if only to guide future research on the subject.

### Implications for Seismic Exploration

One of the most remarkable aspects of these results are the low levels of sound at which a change in calling behavior was detected. Two recent studies, Risch *et al*. [13] and Melcón *et al*. [14], also made that observation regarding their own results. Melcón *et al*. [14], working on the vocal response of foraging blue whales to an MFA sonar sound source, state “It is remarkable that relatively low intensity sound levels cause a perturbation such that the probability of D calls decreases compared to our reference (non-anthropogenic noise). This suggests that a single MFA sonar source could elicit a response from blue whales over a broad region of the Southern California Bight.” Similarly, the area around a seismic ship within which our results predict a behavioral response by bowhead whales is sizable. Based on the SSC data presented in Table 6, calling by bowhead whales is repressed within a radius of ~50–100 km from the seismic ship (~7850–31,410 km^2^), assuming the seismic source and propagation conditions are similar to this study. Within ~10–40 km of such a seismic source (~314–5026 km^2^), calling by bowhead whales would be almost nonexistent. Therefore, under the source and propagation conditions analyzed here, in relatively shallow water (<100 m), we must conclude that monitoring for the presence of migrating bowhead whales within ~40 km of seismic exploration activities cannot be done acoustically.

In the Risch *et al*. [13] study on humpback whales, mentioned above, concurrent visual observations confirmed that male humpback whales were present in the area even when no songs were detected. The authors therefore suggested that male humpbacks may have ceased singing and remained in the area. Similarly, we have no evidence that decreases in calling rates were the result of whales moving away. In a few cases, shut-down of the airguns led to a resumption of calling that was fast enough that the whales must have remained in the area. Cessation of calling is likely one of the first measurable behavioral changes when a whale encounters a sound source such as an airgun array, and calling cessation very likely precedes deflection. It follows that deflection to seismic operations would be a challenge to study in bowhead whales using passive acoustics. In a review of the effects of seismic exploration on bowhead whales, Richardson and Malme [56] state that beyond a distance of ~7.5 km from an airgun array, bowheads rarely show deflection, even though avoidance may occasionally occur at distances of 20 km or more [57]. Changes in surfacing-respiration-dive (SRD) cycles have also been observed and described in a number of studies [11, 58, 12]. Changes in SRD cycles are known to occur at greater distances than deflection, out to ~70 km in some studies [59].

In conclusion, this study has shown an unexpectedly complex change in bowhead whale calling behavior—first an increase, followed by a plateau, and then a decrease—to received levels from airgun sounds. Proximate effects on the animals of such a change in behavior could be minor, but are unknown. Nevertheless, the Bering-Chukchi-Beaufort Sea (BCB) population of bowhead whales has shown a healthy increase in numbers since the late 1970s [60] despite being exposed to airgun pulses since 1968, when MMS issued the first permits for seismic survey activities in the Beaufort Sea [49]. On a global scale, seismic exploration activities are likely to increase in the Arctic, including in areas that are part of the bowhead range but have not been prospected much in the past, such as in the North Atlantic east and west of Greenland. The results presented in this paper will be important in understanding the effects of seismic operations on several scales. On a small scale, they will help in interpreting bowhead calling rates recorded by passive acoustic systems. On a larger scale, they will contribute to the current attempts to better understand lifetime sound exposure of these long-lived, highly migratory marine mammals.

## Supporting Information

S1 Table Deployment coordinates and water depth for 42 DASAR locations used in this study. (DOCX)

S2 Table Information regarding airgun pulses detected at each seven-DASAR array in 2007–2010. (DOCX)

S3 Table Poisson regression model parameter point estimates and bootstrap confidence intervals. (DOCX)

S1 File Further information on false detection rates in automated bowhead call detection routines. (DOCX)

S2 File Section A (Justification for analysis cells of radius 2 km) and Figure A in S2 File. (DOCX)

S3 File Finite-impulse response bandpass filter used in airgun pulse detections. (DOCX)

S4 File Block bootstrapping and use of Poisson regression. (DOCX)

S5 File Section B (Relationship between CSEL thresholds and pulse SPLs) and Figure B in S5 File. (DOCX)
