# Supplementary material for: Effects of Airgun Sounds on Bowhead Whale Calling Rates: Evidence for Two Behavioral Thresholds
Source: PLoS One. 2015 Jun 3;10(6):e0125720. doi: 10.1371/journal.pone.0125720 (PMC4454580; doi:10.1371/journal.pone.0125720)
Supplement: S1 Table — (DOCX) [file pone.0125720.s001.docx]

**S1 Table.** **Deployment coordinates and water depth for 42 DASAR locations used in this study.** Deployment coordinates are in decimal degrees (WGS 84) and the values shown are mean locations across all years (2007–2010). Deployment locations generally varied by no more than a few tens of meters between years.

|  | **Lat. (°)** | **Long. (°)** | **Depth (m)** |  | **Lat. (°)** | **Long. (°)** | **Depth (m)** |
| --- | --- | --- | --- | --- | --- | --- | --- |
| **Site 1** |  |  |  | **Site 3 (cont.)** | |  |  |
| A | 70.7920 | -150.6566 | 20.8 | C | 70.4492 | -146.8028 | 32.7 |
| B | 70.8235 | -150.4913 | 23.1 | D | 70.4790 | -146.6412 | 36.9 |
| C | 70.8551 | -150.6572 | 22.2 | E | 70.5123 | -146.8038 | 37.7 |
| D | 70.8864 | -150.4910 | 24.1 | F | 70.5437 | -146.6396 | 38.2 |
| E | 70.9179 | -150.6562 | 20.8 | G | 70.5750 | -146.8038 | 38.8 |
| F | 70.9493 | -150.4907 | 22.9 | **Site 4** |  |  |  |
| G | 70.9813 | -150.6549 | 16.2 | A | 70.2510 | -145.7218 | 26.7 |
| H | 70.6723 | -150.2594 | 16.2 | B | 70.2823 | -145.5607 | 32.3 |
| I | 70.7282 | -150.1683 | 18.9 | C | 70.3140 | -145.7225 | 31.9 |
| J | 70.6724 | -150.0743 | 15.2 | D | 70.3453 | -145.5603 | 33.7 |
| K | 70.7284 | -149.9843 | 18.7 | E | 70.3769 | -145.7236 | 34.9 |
| L | 70.6724 | -149.8896 | 14.6 | F | 70.4080 | -145.5584 | 38.7 |
| **Site 2** |  |  |  | G | 70.4398 | -145.7222 | 39.7 |
| A | 70.6381 | -148.9504 | 21.2 | H | 70.4085 | -145.8852 | 36.2 |
| B | 70.6695 | -148.7859 | 22.7 | I | 70.3459 | -145.8844 | 31.9 |
| C | 70.7013 | -148.9512 | 24.9 | **Site 5** |  |  |  |
| D | 70.7322 | -148.7871 | 26.8 | A | 70.2466 | -143.3146 | 37.1 |
| E | 70.7640 | -148.9515 | 26.3 | B | 70.2774 | -143.1558 | 46.1 |
| F | 70.7954 | -148.7857 | 32.6 | C | 70.3093 | -143.3155 | 47.3 |
| G | 70.8268 | -148.9518 | 32.0 | D | 70.3407 | -143.1532 | 52.3 |
| **Site 3** |  |  |  | E | 70.3726 | -143.3147 | 53.0 |
| A | 70.3861 | -146.8019 | 27.9 | F | 70.4040 | -143.1525 | 52.4 |
| B | 70.4178 | -146.6409 | 32.9 | G | 70.4353 | -143.3148 | 52.3 |
