# Supplementary material for: Effects of Airgun Sounds on Bowhead Whale Calling Rates: Evidence for Two Behavioral Thresholds
Source: PLoS One. 2015 Jun 3;10(6):e0125720. doi: 10.1371/journal.pone.0125720 (PMC4454580; doi:10.1371/journal.pone.0125720)
Supplement: S2 Table — (DOCX) [file pone.0125720.s002.docx]

**S2 Table. Information regarding airgun pulses detected at each seven-DASAR array in 2007–2010.** (a) Total number of pulses detected at each site. Note this total is the sum of the detections at each DASAR of the site, and is therefore an overestimate of the actual number of airgun pulses present since a specific pulse is likely detected at more than one DASAR. (b) Average number of pulses per functional DASAR. (c) Percentage of pulses that were overloaded. (d) Highest airgun pulse count at each site and identity of the DASAR. In 68% of cases the DASAR that detected the most airgun pulses was at location F or G, at the northern end of the arrays, in deeper water. (e) Median sound pressure level (SPL, in dB re 1 µPa) of received pulses. (f) Median sound exposure level (SEL, in dB re 1 µPa^2^-s) of received pulses.

| **Site** |  | **Year** | **2007** | **2008** | **2009** | **2010** |
| --- | --- | --- | --- | --- | --- | --- |
| **1** | (a) | Total # pulses for site | 8,967 | 492,629 | 36,398 | 36,212 |
|  | (b) | Average per DASAR | 1,793 | 82,105 | 5,200 | 5,173 |
|  | (c) | % overloaded pulses | 0.0% | 32.1% | 0.0% | 0.0% |
|  | (d) | Highest DASAR total (ID) | 6,316 (F) | 101,230 (G) | 9,018 (E) | 23,832 (A) |
|  | (e) | Median SPL | 81.6 | 118.0 | 80.2 | 111.0 |
|  | (f) | Median SEL | 80.6 | 113.0 | 80.4 | 101.5 |
| **2** | (a) | Total # pulses for site | 227,774 | 902,438 | 232,625 | 63,419 |
|  | (b) | Average per DASAR | 32,539 | 128,920 | 33,232 | 31,710 |
|  | (c) | % overloaded pulses | 1.7% | 0.1% | 0.0% | 0.0% |
|  | (d) | Highest DASAR total (ID) | 55,609 (F) | 187,078 (G) | 46,623 (F) | 35,229 (F) |
|  | (e) | Median SPL | 103.2 | 97.1 | 91.9 | 94.2 |
|  | (f) | Median SEL | 102.9 | 97.3 | 92.0 | 95.6 |
| **3** | (a) | Total # pulses for site | 683,125 | 969,244 | 460,034 | 580,555 |
|  | (b) | Average per DASAR | 97,589 | 138,463 | 65,719 | 96,759 |
|  | (c) | % overloaded pulses | 17.6% | 18.7% | 0.0% | 0.1% |
|  | (d) | Highest DASAR total (ID) | 105,336 (E) | 168,555 (G) | 79,058 (G) | 124,906 (G) |
|  | (e) | Median SPL | 123.9 | 124.8 | 100.6 | 106.1 |
|  | (f) | Median SEL | 125.3 | 126.9 | 101.4 | 107.3 |
| **4** | (a) | Total # pulses for site | 597,991 | 807,516 | 393,501 | 644,802 |
|  | (b) | Average per DASAR | 85,427 | 115,359 | 56,214 | 92,115 |
|  | (c) | % overloaded pulses | 39.0% | 35.6% | 0.0% | 0.0% |
|  | (d) | Highest DASAR total (ID) | 94,260 (G) | 141,942 (G) | 69,453 (G) | 128,528 (G) |
|  | (e) | Median SPL | 134.6 | 134.8 | 97.2 | 102.8 |
|  | (f) | Median SEL | 134.9 | 135.6 | 97.8 | 103.7 |
| **5** | (a) | Total # pulses for site | 646,031 | 1,433,876 | 635,884 | 1,103,249 |
|  | (b) | Average per DASAR | 92,290 | 204,839 | 90,841 | 183,875 |
|  | (c) | % overloaded pulses | 0.0% | 0.0% | 0.0% | 0.0% |
|  | (d) | Highest DASAR total (ID) | **106,558 (B)** | **219,392 (D)** | **106,134 (B)** | **195,972 (G)** |
|  | (e) | Median SPL | 103.0 | 105.3 | 103.4 | 109.0 |
|  | (f) | Median SEL | 104.2 | 106.7 | 103.6 | 108.5 |
| Sum of highest DASAR total in each year (all at site 5, in bold): | | | | | | 628,056 |
| Overall total number of detections at all DASARs, all years (sum of all (a) cells): | | | | | | 10,956,212 |
