# Supplementary material for: Effects of Airgun Sounds on Bowhead Whale Calling Rates: Evidence for Two Behavioral Thresholds
Source: PLoS One. 2015 Jun 3;10(6):e0125720. doi: 10.1371/journal.pone.0125720 (PMC4454580; doi:10.1371/journal.pone.0125720)
Supplement: S3 Table — (DOCX) [file pone.0125720.s003.docx]

**S3 Table. Poisson regression model parameter point estimates and bootstrap confidence intervals.** Information is shown for cell-time interval durations of 5 min, 10 min, and 20 min. L95 and U95 represent the lower and upper 95% confidence limits, respectively.

| **Time interval** | **Parameter** | **Interpretation** | **Pt estimate** | **L95** | **U95** |
| --- | --- | --- | --- | --- | --- |
| **5 min** | *β*_0_ | Intercept (Site 3) | -2.57 | -2.72 | -2.41 |
|  | *β*_1_ | Site 1 | -0.965 | -1.286 | -0.684 |
|  | *β*_2_ | Site 2 | 0.564 | 0.295 | 0.812 |
|  | *β*_3_ | Site 4 | 0.177 | -0.028 | 0.371 |
|  | *β*_4_ | Site 5 | 0.280 | 0.097 | 0.471 |
|  | *β*_5_ | Lower thresh. slope | -0.023 | -0.110 | 0.010 |
|  | *β*_6_ | Upper thresh. slope | -0.108 | -0.128 | -0.083 |
|  | Δ_1_ | Lower threshold | 92.0 | 85.8 | 120.7 |
|  | Δ_2_ | Upper threshold | 124.6 | 120.1 | 127.3 |
| **10 min** | *β*_0_ | Intercept (Site 3) | -1.85 | -1.99 | -1.69 |
|  | *β*_1_ | Site 1 | -0.975 | -1.316 | -0.671 |
|  | *β*_2_ | Site 2 | 0.543 | 0.311 | 0.788 |
|  | *β*_3_ | Site 4 | 0.164 | -0.041 | 0.371 |
|  | *β*_4_ | Site 5 | 0.266 | 0.079 | 0.443 |
|  | *β*_5_ | Lower thresh. slope | -0.035 | -0.182 | -0.009 |
|  | *β*_6_ | Upper thresh. slope | -0.106 | -0.126 | -0.079 |
|  | Δ_1_ | Lower threshold | 94.5 | 87.6 | 100.8 |
|  | Δ_2_ | Upper threshold | 127.4 | 122.9 | 129.9 |
| **20 min** | *β*_0_ | Intercept (Site 3) | -1.14 | -1.29 | -0.97 |
|  | *β*_1_ | Site 1 | -0.984 | -1.300 | -0.717 |
|  | *β*_2_ | Site 2 | 0.511 | 0.255 | 0.741 |
|  | *β*_3_ | Site 4 | 0.148 | -0.055 | 0.352 |
|  | *β*_4_ | Site 5 | 0.249 | 0.057 | 0.439 |
|  | *β*_5_ | Lower thresh. slope | -0.037 | -0.148 | -0.018 |
|  | *β*_6_ | Upper thresh. slope | -0.103 | -0.122 | -0.076 |
|  | Δ_1_ | Lower threshold | 97.1 | 87. 8 | 105.2 |
|  | Δ_2_ | Upper threshold | 130.5 | 125. 7 | 132.4 |
