# Supplementary material for: Effects of Airgun Sounds on Bowhead Whale Calling Rates: Evidence for Two Behavioral Thresholds
Source: PLoS One. 2015 Jun 3;10(6):e0125720. doi: 10.1371/journal.pone.0125720 (PMC4454580; doi:10.1371/journal.pone.0125720)
Supplement: S1 File — (DOCX) [file pone.0125720.s004.docx]

**S1 File. Further information on false detection rates in automated bowhead call detection routines.**

The automated detection algorithm has been thoroughly tested against manual analyses of the same data set over a four-year period, a process detailed in [22]. It was found that if the automated call detection parameters were configured so that up to 20% of manually detected calls were missed by the algorithm, between 20 and 40% of all calls detected by the automated algorithm did not correspond with a manual detection. Had the manually-analyzed data set been “perfect,” in the sense that every whale call present in the data had been marked by a manual analyst, then the effective false detection rate would be between 20 and 40%, a very high value. However, as discussed in great detail in Section VI.C in [22], it became clear that the manually analyzed data sets were far from perfect. Biases existed in how many DASARS a given analyst used to measure a call, and when these so-called “false alarms” were plotted spatially against manually-detected call distributions, the overlap was very similar. It was found that 75% of so-called false detections were actually legitimate whale calls; analysts tended not to flag weaker call detections from more distant DASARs. We thus estimate that a 20% miss rate corresponds to a 10 to 20% false detection rate (or precision of 0.8 to 0.9). The large-scale spatial distributions of both the automated and manually analyzed data sets were very consistent, giving further credence to this estimate.
