# Supplementary material for: Effects of Airgun Sounds on Bowhead Whale Calling Rates: Evidence for Two Behavioral Thresholds
Source: PLoS One. 2015 Jun 3;10(6):e0125720. doi: 10.1371/journal.pone.0125720 (PMC4454580; doi:10.1371/journal.pone.0125720)
Supplement: S2 File — (DOCX) [file pone.0125720.s005.docx]

**S2 File. Section A (Justification for analysis cells of radius 2 km) and Figure A.**

# The size of the analysis cells (radius 2 km) was initially chosen based on previous investigations, e.g., [19]. For the purpose of the study presented here, it was important to demonstrate that restricting the samples to calls localized within 2 km of DASARs eliminated the negative relationship between call localization rates and background levels. This is shown below in Fig. A. For every 10-min period throughout the four field seasons (roughly mid-August to early October of 2007–2010, see Table 1), the number of calls localized by each DASAR was plotted as a function of background levels (see below). All localized calls were included in Fig. A(a), whereas only calls localized within 2 km of DASAR locations were included in Fig. A(b). Quantile regression was then used to estimate the 95^th^ percentile of the call distribution, in both plots.

Background levels were computed as follows: for each DASAR, narrowband spectral densities (1 Hz intervals, 1.7 Hz bandwidth, 23.5% overlap) were determined for a one-min period every 4.37 min (262 s). To derive each of these one-min spectra, a series of 119 one-second-long data segments, overlapped by 50% and thus spanning one min, were analyzed. The 119 resulting 1-Hz spectra were averaged to derive a single spectrum documenting narrowband levels for the one-min period. Broadband levels were derived from the narrowband data by summing the mean square pressures of all frequencies within the 10–450 Hz frequency range.

If the start time for any given background measurement (as described above) fell within a pre-defined 10-min period, then the value was assigned to the period, irrespective of whether or not the entire min fell within the period.  Thus, there were either two or, less frequently, three sound values within each 10-min period.  In either case, the set of values was transformed to the linear scale, averaged, and then back-transformed to the dB scale. To minimize the influence of sparse data in the tails of the distribution, only the central 96% of background values were included, i.e., the range 79.1–132.6 dB re 1 μPa. The distribution of the background levels over all four years is shown in Fig. A(c). About sixty percent of background levels were in the range 95–110 dB re 1 μPa.


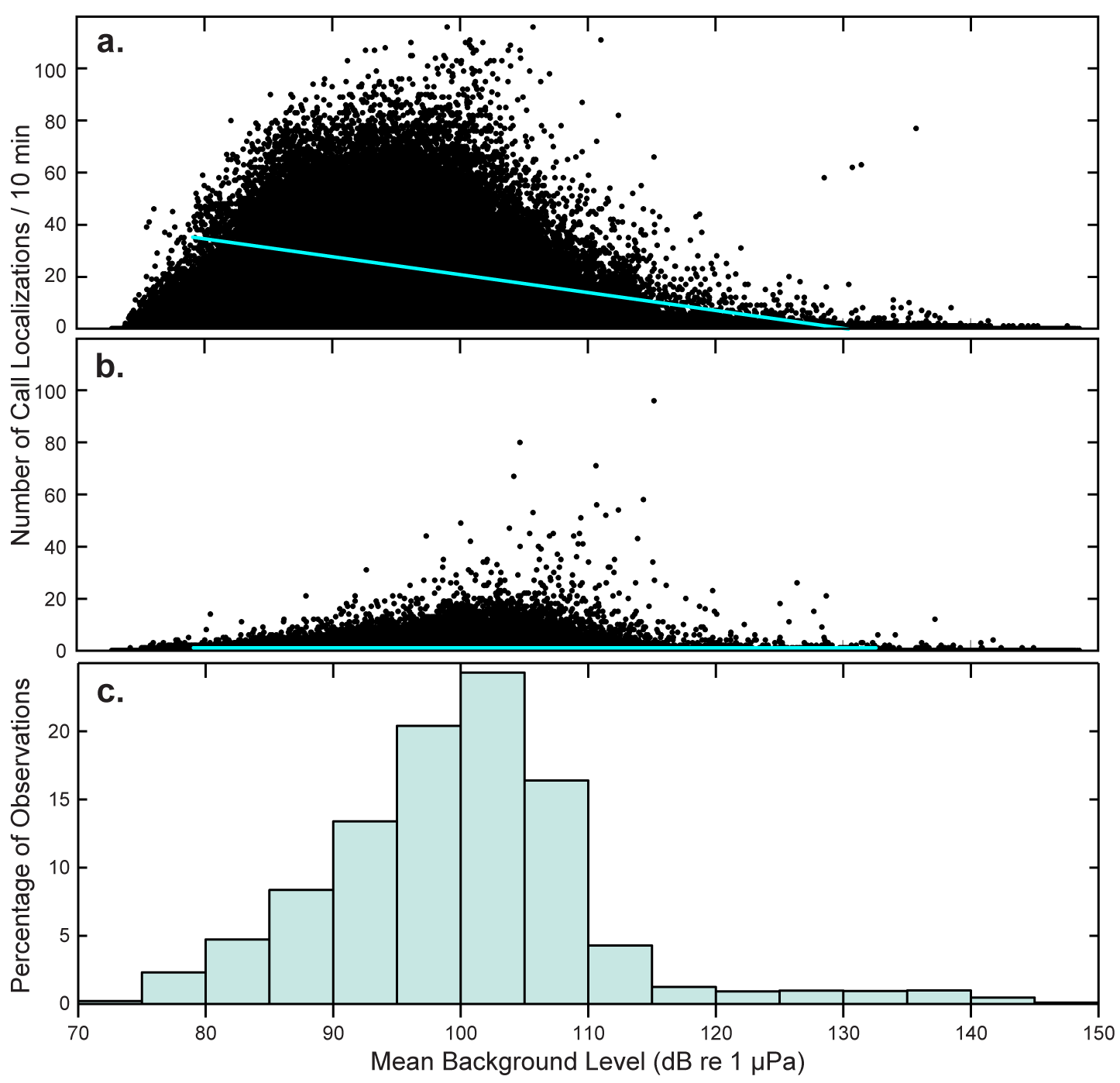


**Fig. A.** **Relationship between background levels and call localization rates.** All localized calls are shown in (a), while (b) only considers calls localized within 2 km of a DASAR. The data shown include call localizations at all DASARs combined over the four field seasons (mid-August to early October 2007–2010). Turquoise lines in (a) and (b) represent the predicted 95^th^ percentile of number of calls based on quantile regressions. (c) Distribution of background levels over the four years at all DASARs combined.

The slope of the quantile regression is -0.6856 for all calls (Fig. A(a), 95% C.I. ‑0.6945, ‑0.6801). This slope indicates that call rate decreases rapidly with increases in background sound, and the associated 95% confidence interval indicates the slope is significantly less than 0. For calls within 2 km of the DASARs (Fig. A(b)), the slope is ‑1.26 x 10^-11^ (95% C.I. ‑2.11 x 10^-10^, ‑9.5 x 10^-14^). Even though the confidence interval does not include 0, this slope is for all practical purposes not different from 0. For example, if background levels increased from 70 to 150 dB re 1 μPa (the entire width of our background data set), the predicted 95^th^ percentile of number of calls detected in 10 min would decrease by approximately 1 x 10^-9^. For comparison, the regression based on all calls predicts a decrease of 13.5 calls per 10 min over the same background level range (70 to 150 dB).
