# Supplementary material for: Effects of Airgun Sounds on Bowhead Whale Calling Rates: Evidence for Two Behavioral Thresholds
Source: PLoS One. 2015 Jun 3;10(6):e0125720. doi: 10.1371/journal.pone.0125720 (PMC4454580; doi:10.1371/journal.pone.0125720)
Supplement: S3 File — (DOCX) [file pone.0125720.s006.docx]

# S3 File. Finite-impulse response bandpass filter used in airgun pulse detections.

# The finite-impulse response (FIR) bandpass filter was customized for each airgun pulse, to account for the fact that the airgun signal bandwidth could vary depending on source range, source type, and signal-to-noise ratio (SNR). The lower cutoff frequency was fixed at 10.5 Hz (with a 1 Hz transition band from 10 to 11 Hz). This lower cutoff was selected in order to remove low-frequency energy arriving from bottom interface waves and head waves, which are often detectable when the airgun survey was within a few tens of km from a given DASAR. The upper cutoff frequency was customized for each detected pulse by estimating the SNR of the detected pulse over overlapping 50 Hz bandwidth segments on a spectrogram. Bandwidth measurements with SNR values below 6 dB were interpreted as indicating the pulse was not present in that given bandwidth. The highest 50 Hz bandwidth region that passed this SNR test was set to be the upper cutoff frequency of the customized FIR filter. For further details on how the SNR of a transient detection was computed as a function of frequency, please see the discussion about the first stage of the automated bowhead whale call detector in [22].
