# Supplementary material for: Effects of Airgun Sounds on Bowhead Whale Calling Rates: Evidence for Two Behavioral Thresholds
Source: PLoS One. 2015 Jun 3;10(6):e0125720. doi: 10.1371/journal.pone.0125720 (PMC4454580; doi:10.1371/journal.pone.0125720)
Supplement: S4 File — (DOCX) [file pone.0125720.s007.docx]

# S4 File. Block bootstrapping and use of Poisson regression.

The bootstrap procedure was carried out as follows. (1) For each bootstrap iteration a starting time was randomly generated within the 6-hour period preceding the first observation (cell-time interval with qualifying *CSEL_t_* value). (2) Sequential non-overlapping 6-hour blocks of time were defined such that all observations were assigned to a block. (3) For each site, a new time series was constructed by sampling blocks with replacement. (4) The Poisson regression model (Eq. 2) was re-fitted to the newly constructed dataset, and the resulting parameter estimates were stored. (5) One thousand block bootstrap iterations were completed to compute the distribution means, and the 95% confidence intervals using the percentile method.

The block size of 6 hours was chosen because previous studies (McDonald et al. 2012) found that autocorrelation in calling rates generally diminished to 0 after 6 to 12 hours. The bootstrap method accounted for potential autocorrelation in the time series of call counts at a site and thereby reduced the effective sample size. In addition, the bootstrap procedure accounted for potential extra-Poisson variation in whale call counts. For instance, the large number of zero-counts and occasional very large counts both may have led to over-dispersion. In a more conventional setting, the additional variance would have been better represented by either an over-dispersed Poisson or a Negative Binomial model. Regarding our choice of the Poisson, we note: (1) the standard Poisson distribution has the advantage of relative simplicity, particularly in the context of a complex threshold model that presented its own estimation challenges; (2) the Poisson and Negative Binomial models often yield very similar parameter estimates, as they did for our static (non-bootstrapped) threshold model; and (3) estimates of parameter variances were obtained from the bootstrap distributions and did not rely on Poisson model asymptotics. In short, given our overall modeling approach, use of the Poisson was appropriate, and an alternative model that directly accounted for over-dispersed data would have offered no advantages.

McDonald TL, Richardson WJ, Greene CR Jr, Blackwell SB, Nations CS, Nielson RM, et al. Detecting changes in distribution of calling bowhead whales exposed to fluctuating anthropogenic sounds. J Cetacean Res Manag. 2012;12(1):91–106.
