# Supplementary material for: Effects of Airgun Sounds on Bowhead Whale Calling Rates: Evidence for Two Behavioral Thresholds
Source: PLoS One. 2015 Jun 3;10(6):e0125720. doi: 10.1371/journal.pone.0125720 (PMC4454580; doi:10.1371/journal.pone.0125720)
Supplement: S5 File — (DOCX) [file pone.0125720.s008.docx]

# S5 File. Section B (Relationship between CSEL thresholds and pulse SPLs) and Figure B.

For marine mammal mitigation during seismic operations in the Alaskan Arctic since 1995 (Southall et al. 2007), do-not-exceed criteria have been based on sound pressure levels (SPL), in dB re 1 µPa (NMFS 2000, Allen 2004). Fig. B was therefore generated to facilitate interpreting the cumulative sound exposure levels presented. The relationship between SPL and SEL (and therefore *CSEL_10-min_*) will depend on the airgun pulse duration, which generally increases with distance from the airgun array. Fig. B shows, for five different airgun pulse lengths, the relationship between *CSEL_10-min_* and the corresponding received single pulse SPL. Again, we assume sixty airgun pulses over 10 minutes (i.e., no airgun pulses from other ships) and a constant pulse amplitude. For example, if the pulse length is 0.5 sec, a *CSEL_10-min_* of ~127 dB re 1 µPa^2^-s corresponds to a received single pulse SPL of ~112 dB re 1 µPa. Similarly, a *CSEL_10-min_* of 160 dB re 1 µPa^2^-s corresponds to a single pulse SPL of ~153 dB re 1 µPa when pulse duration is very short, 0.08 s.


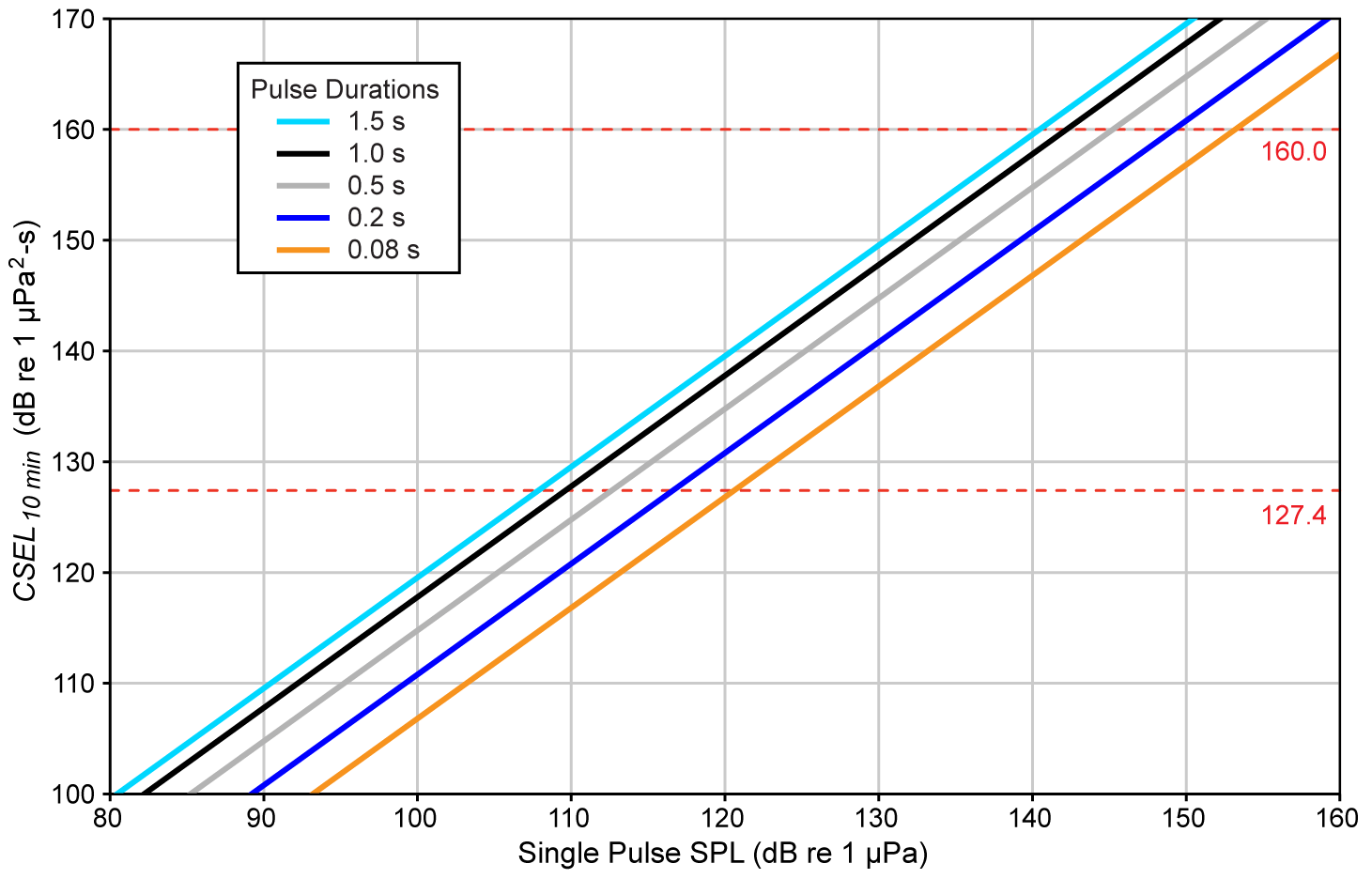


**Fig. B.** **Relationship between *CSEL_10-min_* and single pulse SPL as a function of airgun pulse duration.** The calculations assume 60 airgun pulses of the same amplitude in a 10-min period (a shot every 10 s, corresponding to the firing rate of the *Gilavar*). Two *CSEL_10-min_* levels are shown in dashed lines: the upper threshold of 127.4 dB re 1 µPa^2^-s, where calling rates start decreasing, and 160 dB, a level at which very few bowhead calls were detected (see Fig. 9).

Allen LK. Fed Regist. 2004;69:67535–67539.

NMFS.  Small takes of marine mammals incidental to specified activities; marine seismic-reflection data collection in southern California/Notice of receipt of application.  Fed Regist. 2000;65:16374–16379.

Southall BL, Bowles AE, Ellison WT, Finneran JJ, Gentry RL, Greene CR Jr, et al. Marine mammal noise exposure criteria: initial scientific recommendations. Aquat Mamm. 2007;33:411–521.
